# Animal models to study acute and chronic intestinal inflammation in mammals

**DOI:** 10.1186/s13099-015-0076-y

**Published:** 2015-11-10

**Authors:** Janelle A. Jiminez, Trina C. Uwiera, G. Douglas Inglis, Richard R. E. Uwiera

**Affiliations:** Agriculture and Agri-Food Canada Research Centre, Lethbridge, AB Canada; Department of Agricultural, Food and Nutritional Science, University of Alberta, Edmonton, AB Canada; Department of Surgery, Faculty of Medicine and Dentistry, University of Alberta, Edmonton, AB Canada

**Keywords:** Intestine, Inflammation, Acute, Chronic, Animal models, Incitants, Chemical, Biological

## Abstract

Acute and chronic inflammatory diseases of the intestine impart a significant and negative impact on the health and well-being of human and non-human mammalian animals. Understanding the underlying mechanisms of inflammatory disease is mandatory to develop effective treatment and prevention strategies. As inflammatory disease etiologies are multifactorial, the use of appropriate animal models and associated metrics of disease are essential. In this regard, animal models used alone or in combination to study acute and chronic inflammatory disease of the mammalian intestine paired with commonly used inflammation-inducing agents are reviewed. This includes both chemical and biological incitants of inflammation, and both non-mammalian (i.e. nematodes, insects, and fish) and mammalian (i.e. rodents, rabbits, pigs, ruminants, dogs, and non-human primates) models of intestinal inflammation including germ-free, gnotobiotic, as well as surgical, and genetically modified animals. Importantly, chemical and biological incitants induce inflammation via a multitude of mechanisms, and intestinal inflammation and injury can vary greatly according to the incitant and animal model used, allowing studies to ascertain both long-term and short-term effects of inflammation. Thus, researchers and clinicians should be aware of the relative strengths and limitations of the various animal models used to study acute and chronic inflammatory diseases of the mammalian intestine, and the scope and relevance of outcomes achievable based on this knowledge. The ability to induce inflammation to mimic common human diseases is an important factor of a successful animal model, however other mechanisms of disease such as the amount of infective agent to induce disease, invasion mechanisms, and the effect various physiologic changes can have on inducing damage are also important features. In many cases, the use of multiple animal models in combination with both chemical and biological incitants is necessary to answer the specific question being addressed regarding intestinal disease. Some incitants can induce acute responses in certain animal models while others can be used to induce chronic responses; this review aims to illustrate the strengths and weaknesses in each animal model and to guide the choice of an appropriate acute or chronic incitant to facilitate intestinal disease.

## Background

Diseases are often categorized by the organs affected and the resulting clinical manifestations produced. Inflammation is a collection of conserved immunological processes that lead to the recovery and repair of damaged tissue, with the potential to cause more damage and harm when insufficiently regulated. In the intestine, controlled inflammation is necessary for immunological function, as regulatory immune cells are continually interacting with intestinal bacteria and food particles to regulate pro-inflammatory effector cells and facilitate anti-inflammatory pathways [1]. Events such as epithelial barrier disruptions, uncontrolled bacterial colonization, unregulated immune effector cell stimulation and the dysregulation of the homeostatic balance can contribute to disease onset. Furthermore, these events can manifest anywhere in the small intestine (i.e. duodenum, jejunum, ileum) and/or large intestine (i.e. cecum, appendix, colon, rectum) [2, 3]. Disease duration is also an important factor in characterizing intestinal disease with chronic diseases persisting for months or longer, and acute diseases lasting only weeks, typically from 7 to 14 days [4, 5].

Acute and chronic inflammatory diseases of the intestine induce a number of health-related problems, and decrease the quality of life in people in both developing and developed countries [6, 7]. Diarrhea is a common presentation of intestinal enteritis, and nearly 1.7 billion cases of diarrheal disease are reported globally each year [8], however cases of enteritis often go unreported due to their self-limiting nature [9]. In North America and Australia, the prevalence of acute enteritis incited by foodborne pathogens alone is estimated to affect approximately 10–20 % of the human population annually [9]. Importantly, acute enteritis or diarrheal disease is the second leading cause of death worldwide, accounting for over 1 million deaths annually [10] with higher mortality rates in developing countries [11]. Acute enteritis also imparts significant direct and indirect costs to society, including lost worker productivity and direct impacts on health care systems. In Canada for example, intestinal disease is responsible for the hospitalization of 4 % of children ≤5 years of age [12]. In the United States, the Center for Disease Control reported that costs for hospitalization, emergency room visits and outpatient care for children with viral-induced acute enteritis averaged $273 million from 2009 to 2010 [13], while in Canada in recent years, acute enteritis incited by viruses was estimated at $20 million dollars in hospitalization costs for older individuals [14]. Large numbers of people also suffer from chronic inflammatory diseases of the intestine, and chronic enteritis rates continue to rise [15, 16]. Inflammatory bowel disease (IBD) is the most important chronic inflammatory disease of people, and it includes Crohn’s disease (CD) and ulcerative colitis (UC) [16]. In Canada approximately 0.7 % of the population were living with inflammatory bowel disease in 2012 [17, 18], and diagnosis and treatment costs to the health care system for Canadians afflicted with CD or UC was estimated at $1.2 billion [18]. Other developed nations such as Europe and Australia also have a high prevalence of IBD [19, 20], and rates in Asian countries are increasing [15]. Chronic inflammatory diseases of the intestine have a tremendous negative impact on the health and well-being of individuals and costs to health care systems.

**Fig. 1:**
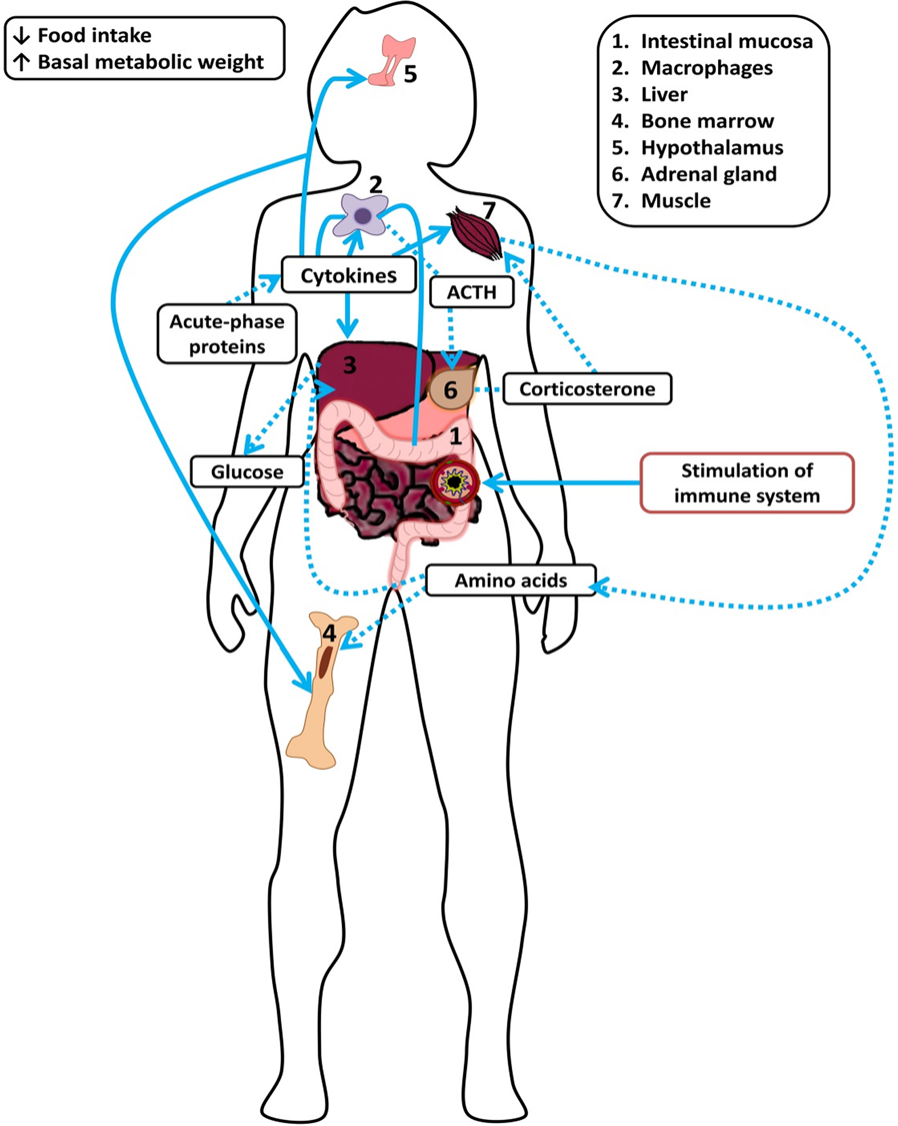
Systemic effects of intestinal inflammation. Multiple areas of the body can be influenced by intestinal inflammation. After stimulation occurs in the intestine, circulating macrophages stimulate the release of cytokines. These cytokines not only promote macrophage and dendritic cell recruitment, but also stimulate the hypothalamus to alter food intake and increase the rate of metabolic activity through adrenal gland stimulation by ACTH and the production of corticosterone. Simultaneously, cytokines stimulate muscles to promote amino acid usage that can influence the liver and bone marrow. Acute-phase proteins released by the liver also influence other cytokine production, and the intermingled cycle continues as the intestinal mucosa is stimulated. *ACTH* adrenocorticotropic hormone

Although inflammatory diseases of the intestine are often referenced with regard to their localized and temporal inflammatory effects within the small or large intestine, uncontrolled inflammation of the intestine always imparts a systemic impact on the body [21, 22] (Fig. 1). Significantly, the etiology of both acute and chronic intestinal inflammatory disease is often enigmatic, [16, 23] thereby compromising treatment choices and efficacy. Furthermore, chronic inflammatory diseases of the intestine such as IBD are often linked to prior acute inflammatory disease incited by viruses, bacteria, parasites [24], dysregulation of the intestinal immune response, or autoimmune disorders [25]. The appropriate use of animal models is essential to ascertain the etiology of intestinal inflammatory diseases, and is advantageous when elucidating the processes involved in the onset and progression of acute and chronic disease. Effectively applied animal models are instrumental to the development and prevention of appropriate mitigation strategies. Understanding the limitations, benefits, differences and similarities between various animal models, and the chemical and biological methods that can be used to advance them is essential in the successful mechanistic understanding of disease.

### Intestine and the immune system

**Fig. 2:**
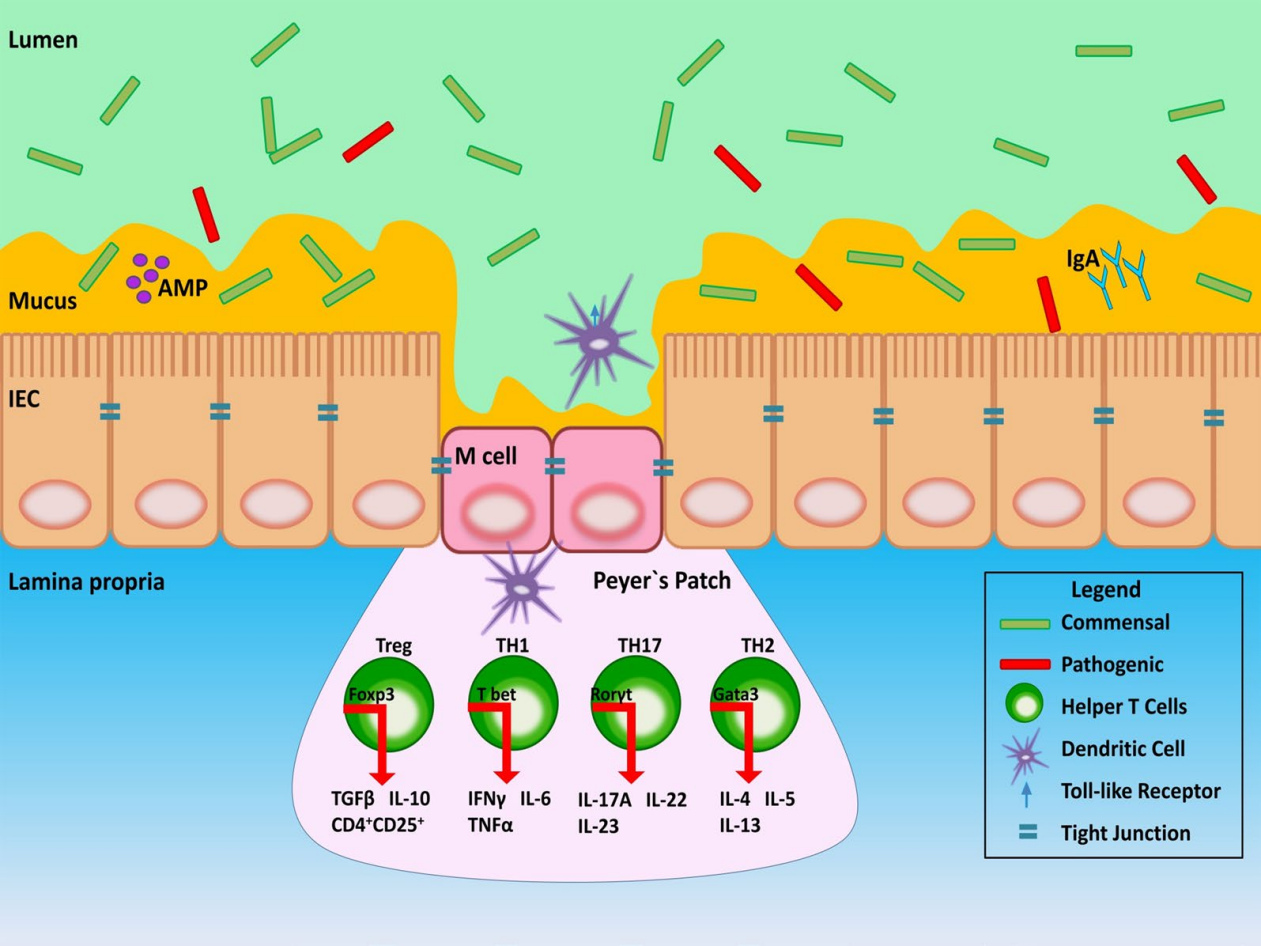
Small intestine mucosal immunity. Intestinal epithelial cells (IEC) make up the epithelial lining. The IEC are covered by mucus which serves as an important component of the innate immune system. In the large intestine mucus is divided into two distinct layers that vary in thickness ; the *thicker outer layer* being bacteria-rich and the *thinner inner layer* containing no or few bacteria  (not shown in image). The epithelium is composed of enterocytes and M cells, and these cells are held together by tight junction proteins. Of note, these cells and structures are also areas where bacteria can enter the host to induce inflammation and activate underlying immune cells. Cells important in immuno-surveillance, such as dendritic cells within the lamina propria can move through M cells or tight junctions in the IECs to sample luminal contents [28]. This information is presented to T-cell populations through the secretion of cytokines to facilitate cell maturation and proliferation [294]. Th1 (IFN-γ, IL-6, TNF-α) cytokines and Th17 (IL-17A, IL-22) cytokines activate pro-inflammatory responses, Treg (IL-10, TGF-β) cytokines are anti-inflammatory and Th2 (IL-4, IL-5, IL-13) cytokines have both anti-inflammatory and pro-inflammatory potential. Immunoglobulin A (IgA) is released from plasma cells at intestinal crypts while antimicrobial peptides (AMP) and defensins are released from Paneth cells (not shown) [28]. *M cell* microfold cell, *TLR* toll-like receptor, *Th* helper T-cell. Transcription factors; Foxp3 (Treg), T bet (Th1), Rorγt (Th17), Gata3 (Th2)

The immune system within the intestine is a complex system; combining coordinated responses between the innate and adaptive immune systems within the intestinal mucosa [26–28]. The innate and adaptive responses are composed of both cellular and non-cellular components (Fig. 2). In the innate response, the non-cellular (humoral) components range from physical (epithelial lining, tight junctions, M cells) and chemical barriers (stomach acid, mucin) to antimicrobial proteins (cryptidins, β-defensin α-defensin, heat shock proteins, compliment), cytokines and chemokines, Toll-like receptors (TLRs), Nod-like receptors (NODs) and enzymes (peptidase, nuclease, lipase), and play a critical role in minimizing the number of infections the immune system encounters [29, 30]. Cellular components of innate immunity include macrophages, mast cells, neutrophils, eosinophils, natural killer (NK) cells, NK T-cells, and dendritic cells, which can engulf and eliminate harmful pathogens [31]. Macrophages, and in particular dendritic cells, also act as antigen presenting cells (APC) which engulf the recognized pathogens and present their antigens to components of the acquired immune system such as T-cells [32]. This process enables the two immune systems to operate in a coordinated manner.

Antibodies are the non-cellular components of the adaptive immune system produced by plasma B-cells and act to bind pathogens [33]. The pathogens are either neutralized by agglutination with antibodies, or are targeted for destruction by the following methods: (1) activation of the compliment system; (2) opsonisation to granulocytes; or (3) release of cytokine cascades for NK cells [30]. The main lymphocytic cellular components of the adaptive immune system are composed of CD8^+^ and CD4^+^ T-cells. CD8^+^ T-cells are responsible for recognizing and destroying organisms, primarily through the release of perforins and granzymes [28]. These function to create pores in the cell lipid bilayer and digest cellular material to promote apoptosis, respectively [28]. Cytotoxic CD8^+^ T-cells can also enhance the release of effector cytokines, such as IFN-γ, TNF-α and TNF-β, which collectively promote macrophage activation and cell death [28]. CD4^+^ T-cells or helper T-cells are important in the coordination of immune and inflammatory responses through the release of cytokines. There are five different subsets of CD4^+^ helper T-cells (Table 1) and the activation of the various helper T-cell responses is often determined by the type of pathogen affecting the host. For example, the Th1 response is mostly associated with infections by intracellular pathogens (i.e. viruses and bacteria), whereas Th2 responses defend against extracellular pathogens such as parasites [27]. Although the immune responses involved in intestinal inflammation are not the main theme of the current article, it is important to consider the components of the innate and adaptive immune response when examining the causes and manifestation of intestinal injury and inflammation.

**Table 1:** Summary of T-cell subsets derived from naïve CD4^+^ T-cells [295]

T-cell subset	Activation factor	Transcription factor	Cytokines produced
Tfh	STAT3	Bcl6	IL-21
Th1	STAT4	T-bet	IFN-γ
Th2	STAT6	Gata3	IL-4, IL-13
Th17	STAT3	Rorγt	IL-17A, IL-17F
Treg	STAT5	Foxp3	IL-10, IL-35, TGF-β

### The intestinal microbiome

Microbial interactions and the intestinal environment are associated with the development and progression of many intestinal diseases. The composition of the intestinal microbiome can be influenced by diet, biological and non-biological agents, host genetics, intestinal injury, infection, host stress and transmission of microorganisms from the vaginal canal during birth [34, 35]. Changes to the species diversity in the intestinal microbiome can affect intestinal inflammation, and studies investigating the addition of probiotic bacteria to the diet have concluded that specific bacteria have the ability to change the metabolic profile to support the growth of carbohydrate-reducing bacteria [36]. These bacteria contribute to the increase of bioactive molecules beneficial for enterocyte growth and development [36]. Furthermore, the addition of probiotic cultures may preferentially bind to the epithelial surface in the intestine and therein compete against and inhibit the attachment and subsequent colonization of pathogenic species, and this in combination with the consumption of prebiotics can also stimulate the growth of commensal bacteria to limit allochthonous  (i.e. non-resident)  bacteria [36]. In both human and non-human animal models, changes in intestinal bacterial communities can affect intestinal disease development [36]. In people, the colonic community is highly diverse within the *Bacteroidetes* and *Firmicutes* phyla, and a loss of diversity in these groups is commonly observed in patients with IBD, while others report an increase in diversity and abundance in bacteria belonging to the *Proteobacteria* phylum during IBD related infections as well as acute enteric infections [37–39]. This shift in abundance of species that utilize carbohydrates for energy to species that utilize proteins for energy illustrates the relationship between metabolic activities of bacteria and intestinal disease [40, 41]. Bacteria in the *Firmicutes* phylum are also important in health maintenance, as many bacteria within the class *Clostridia* have been implicated in immune development and maintenance of intestinal homeostasis [42]. Not only is the presence of specific bacterial groups paramount to the function of the intestine, but the interactions between the resident (i.e. autochthonous) bacterial species and the transient allochthonous bacterial species plays a significant role in disease development. Colonization resistance is the phenomenon whereby the intestinal microbiome protects the host from pathogenic microorganisms [43]. Resident microorganisms have specialized methods that inhibit transient bacterial species that are often pathogenic from occupying specific intestinal niches; the colonization resistance process limits the likelihood of pathogen attachment, growth and damage [44]. Along with the complex microorganism–microorganism interactions, the host has developed methods directed by the innate immune response to mitigate bacterial-induced inflammation in the intestine at the epithelial-microbial interface [45]. Due to the complexity and involvement of highly prescribed processes specific to host-microorganism interactions, it is not possible to simulate the complex and interactive processes that occur within the intestine using in vitro animal models.

### Animal models of inflammation

The intestine is a highly complex organ necessitating sophisticated and comprehensive animal models to study its function and disease. The diverse cell populations within the intestine contribute to this complexity. Additionally, the intestine is in contact with components of food digestion and maintains an environment rich in aerobic, obligate anaerobic, and facultative anaerobic bacteria. These bacteria frequently have modest to marked effects on the intestine and its physiologic and immunologic functions. Identifying mechanisms involved in intestinal injury is necessary to develop mitigation strategies to prevent disease. Studies using tissue samples from affected human beings would provide the most reliable data; however, there are indeed difficulties in acquiring human tissue for research including: the ethical use of collected human tissue; the often small sample size; the pronounced genetic variability between tissue from various individuals; and the isolation of the intestinal tissue from the whole organism that can limit the use of human beings when investigating intestinal disease. As such, comparative whole animal models are needed to provide information representative of intestinal disease in people, and ideally these models provide large sample sizes and have genetically homogeneous backgrounds (i.e. genetically engineered rodent models).

A variety of mammalian models have been used to study acute and chronic intestinal inflammation. Mice are considered a good animal model as their intestinal development is relatively similar to the human intestine and they have many of the same immune responses and genes [46]. Rat models have the advantage of being larger than mice thus allowing the acquisition of larger samples for analysis. Invertebrates, including nematodes and *Drosophila* have also been used in intestinal studies, primarily to investigate mechanisms involved in innate immunity [47]. Zebrafish are extensively used to study both innate and adaptive immune responses [48]. Pigs are commonly used as an alternative monogastric mammalian model, as their intestinal function and morphology is similar to human beings [49]. Non-human primates (NHP) provide the best and most comparable data to people due to their high degree of genetic and physiological similarity to the human intestine. The use of NHPs in research however comes with considerable drawbacks—namely high costs, ethical considerations, and the potential hazards of carrying highly virulent zoonotic agents [50, 51]. Other mammals have also been used to investigate intestinal inflammation including rabbits, guinea pigs [52], and to a lesser degree, ruminants. Dogs develop IBD and exhibit similar gene dysfunction to people with CD, and many studies have used canine models to identify biomarkers of IBD [53, 54]. Collectively, the above models can be used to determine the mechanisms in the development and progression of intestinal disease. Moreover, relatively sophisticated surgical models, such as the intestinal loop technique have been developed to further elucidate mechanisms. Although no single animal model is perfect for studying all components of intestinal inflammation, each possess unique features to explore the various aspects of intestinal injury and disease.

### Mice

Mice are the most commonly used animal model for intestinal studies, and genetically engineered mice are particularly important in studying intestinal inflammation [55]. There are many reviews that summarize and discuss the use of genetically modified murine models to study intestinal disease [56–58], and this section of the review will focus on the advantages and disadvantages of using rodent models. A variety of gene knockout models are available to study innate and adaptive immune responses during intestinal infection (Table 2). Their genetic lines can be modified to produce phenotypes that investigate specific aspects of intestinal inflammation associated with adaptive and innate immune responses, ranging from the activation of proteins involved in pathogen recognition to the activation of effector cells necessary to trigger both cell-mediated and humoral immune responses. Thus, the rapid expansion in the use of genetically modified mice has allowed investigators to study various aspects of intestinal inflammation and disease (Table 2).

**Table 2:** Summary of common knockout genes used in murine models of intestinal inflammation

Genes	Function	Immunity affected	Reference
IL-10	Cytokine involved in anti-inflammatory and regulatory pathways	Adaptive	[57, 64, 296, 297]
IL-23R	Th17 cytokine; T-cell differentiation	Adaptive	[296]
CD4^+^CD25^+^	Regulatory T-cell adaptor glycoproteins	Adaptive	[296, 297]
NOD2/CARD15	Intracellular bacterial peptidoglycan receptor/apoptotic protein	Innate	[296]
TGF-β1	Regulatory cytokine; inhibits effector T-cell development, downregulates immune response	Adaptive	[297]
RAG	Protein; B and T lymphocyte maturation	Adaptive	[57, 64, 296]
ATG16L1	Autophagy gene involved in pathogen regulation	Innate and adaptive	[296]
APC^min/+^	Gene; Β-catenin regulator involved in CRC development	Innate	[64]
IL-2	Pro-inflammatory cytokine	Adaptive	[57]
TNF-α	Th1 cytokine; apoptotic signal activation	Innate and adaptive	[57]
STAT3	Signalling molecule; intestinal mucosa regeneration post injury	Adaptive	[296]
NFκB	Transcription factor; pro-inflammatory cytokines and cell survival factors	Adaptive	[57, 296]
Muc2	Gene; mucin, main constituent of intestinal mucus, physical barrier formation	Innate and adaptive	[296]
IFN-γ	Pro-inflammatory cytokine	Innate and adaptive	[57]
MyD88	Transcription factor; signalling molecule for TLR and NFκB	Innate and adaptive	[64]
TLR	Family of receptors for identification of various microbial surface proteins	Innate	[64]

Certain genetic lines of mice are more suited to investigate specific conditions of acute and chronic enteritis, and it is imperative to choose genetic strains of mice that best represent the aspects of disease being investigated. For example, IL-10 knockout mice with a C3H and BALB/c background are known to develop spontaneous colitis more frequently than wild-type C57BL/6 mice [56]. Alternatively, the C57BL/6 background is more susceptible to Th2 mediated colitis than the BALB/c and C3H/HeJ backgrounds, and TCRα chain deficient C57BL/6 mice are more susceptible to colitis than their BALB/c and C3H/HeJ counterparts [56]. Moreover, it is known that chemical agents used to induce chronic intestinal inflammation can be affected by the genetic background of the mouse. For instance, trinitrobenzene sulfonic acid (TNBS), a chemical used to incite CD-like symptoms in mice, depends largely on genetic background; in SJL/J, C3HeJ and BALB/c mice treated with TNBS, mice develop CD-like intestinal lesions, whereas C57BL/6 mice under the same conditions remain relatively unaffected [56]. Similarly, Swiss Webster and C3H/HeJ mice develop UC-like lesions after the administration of dextran sulphate sodium (DSS), and C57BL/6 mice treated with DSS display less tissue injury [59]. The ability to genetically modify mice, and to a lesser extent rats and other rodents, is a crucial asset for elucidating mechanisms of acute and chronic intestinal inflammation.

The majority of genetically engineered rodent models have been developed solely for research purposes and many knockout models are designed to study the loss of regulatory gene function, or the over stimulation of pro-inflammatory effector molecules (Table 2). Immunocompromised genotypes have also been very useful in the investigation of colitis-inducing immune responses. Immunocompromised mouse models include severe-combined-immunodeficient (SCID) and Rag^−/−^ mice combined with the supplementation of CD4^+^CD45RB^high^ naïve T-cells which lack the ability to produce functional B and T lymphocytes. In these models, the supplemented naive T-cells interact with antigens and become activated as colitogenic T-cells and result in chronic transmural inflammation in both the small and large intestine [60, 61]. Other strategies such as the use of microRNAs to target genes such as tumour suppressor genes have also been suggested as quick and efficient methods to induce colorectal and colitis-associated cancer models [62]. To investigate inflammatory responses triggered by microbial components, germ-free and gnotobiotic mice provide an environment where there is no microbial colonization within the intestine (i.e. axenic), or the intestine is colonized with a relatively small number of defined bacteria such as the Altered Schaedler Flora [63]. Both microbial conditions have proved valuable in elucidating important aspects of host-microorganism interactions [64]. A number of researchers have attempted to create ‘humanized’ mice, which either have human genes knocked into their genomes, or have established a human microbiome in germ-free mice via the transplantation of bacteria within human fecal material to create models potentially more applicable to human diseases [65, 66]. Advancements in genetic engineering and the use of the murine animal model have provided a versatile platform for mice in the elucidation of mechanisms necessary to understand intestinal disease.

Other qualities such as the similarities between the murine and human microbiome, immune responses, as well as the monogastric anatomical structure are important considerations when choosing mice to investigate intestinal injury. Mice share many specific intestinal genes with people, and mapping of the mouse genome and comparative genomic studies concluded that over 90 % of human and mouse genes are shared among the two species, and approximately 80 % of the mouse genes have a human orthologue [46, 67]. Furthermore, human and murine intestinal communities exhibit the same diversity of species within the *Firmicutes*, *Bacteroidetes*, and *Proteobacteria* phyla [68]. The mouse gastrointestinal tract is also anatomically and functionally similar to human beings, and importantly, mice have many features analogous to the adaptive immune response such as the presence of similar populations of B-cells, T-cells, and isotype antibodies [69]. Other salient advantages of mice as an animal model includes their small size (e.g. efficiency of husbandry), their relatively short estrous cycles and gestation period, and their large litter sizes [70].

A broad armamentarium of analytical tools is required to comprehensively investigate intestinal inflammation. Indeed, analysis tools such as monoclonal antibodies, accurately designed PCR primers and cell assays are necessary. There are many commercially available diagnostic biomolecules available to study inflammation in rodents, and rodent models having their entire genome sequenced, possess a large repertoire of available biomolecules and reagents [67, 71]. Many analysis techniques such as cytokine arrays, fluorescence in situ hybridization methods, and sequencing platforms have well developed and quality checked methodologies that work well with tissue isolated from rodents. Using established biomolecules in conjunction with specialized methods and techniques tailored to mice allows researchers to investigate a broad range of detailed and specific cellular processes involved in intestinal inflammation.

Although there are many similarities between mice and human beings inlcuding gene homology, immune and intestinal function, physiology [67], and intestinal bacterial community structure, there are some significant disadvantages to employing mice and other rodent models to study aspects of human intestinal inflammation. Importantly, the intestinal lesions observed in people with IBD are not identical to lesions observed in mice following exposure to chemical agents. For instance, although the administration of DSS to mice induces chemical injury to the epithelial lining which mimics mucosal injury observed in people with UC [72], the severity of the lesions are not consistently representative of human beings with UC. Moreover, there are also differences between the murine and human expression of TLR2, TLR3, TLR4, and TLR9. Murine models exhibit strong mRNA expression patterns of these TLRs in macrophages following exposure to bacterial LPS [73]. In contrast, TLR3 expression in people is restricted to dendritic cells, whereas TLR2 and TLR4 expression are restricted to myelomonocytic cells [73, 74].

Behavioural patterns in mice also impact intestinal disease and can potentially confound the study of diet on the induction and progression of intestinal inflammation. Coprophagy, a nocturnal behaviour in mice, is important for re-ingestion of nutrients and can affect dietary balance, microbial populations, and potentially affect intestinal health [75]. In contrast, coprophagy is not considered a normal behaviour in people, and thus extrapolation of dietary effects on intestinal inflammation in mice to human beings can provide inaccurate interpretations. Another potential disadvantage of using rodent models and specifically mice to study intestinal inflammation is the relatively small sample sizes that can be harvested from mice. Often, this requires that substrates or tissues are pooled from multiple animals for analyses. The necessity of pooling samples requires that multiple animals are used for the study, and this subsequently increases the cost and fails to reduce the numbers of animals required for the research—an important ethical consideration in animal experimentation. The small size of mice can also limit the implementation of surgical procedures, and the use of radiotelemetry devices, endoscopes, and ultrasound equipment [76, 77]. Although there are drawbacks to using mice, the multitude of advantages make mice an excellent choice to investigate processes involved in intestinal inflammation of mammals.

### Rats

 Rats are also frequently employed as an animal model to study intestinal injury and disease. Many of the chemicals used to incite acute inflammation in murine models are also useful in rat models. Interestingly and as an example, the TNBS model was initially developed for rats, and it is currently widely used in other organisms such as mice and zebrafish [78–80]. Furthermore, other chemical incitants such as DSS (a model for IBD) also induces injury to colonic tissue similar to the tissue injury observed in mice [72, 81]. One of the most commonly used genetically modified rat models is the transgenic HLA-B27 model of colitis, which spontaneously develops gastrointestinal inflammation including gastroduodenitis and colitis, as well as arthritis and spondylitis [82, 83]. Finally, an alternate rat model of acute inflammation has also been established using the biological incitant *Campylobacter jejuni*, suggesting that both chemical and biological agents are useful to induce colitis in the rat animal model [84]. Rats are often used in nutritional studies. For example, researchers have utilized the rat model to investigate the impact of fiber-rich diets on intestinal microbial community structure [85, 86]. These groups have investigated changes in community structure observed in feeding trials in rats, and have importantly been able to compare their observations with similar findings in swine. This suggests that nutritional studies in the rat intestine can be compared to other non-rodent monogastric animal models [85].

Although both rats and mice are considered to primarily be cecal fermenters, recent research has shown that a considerable amount of fermentation occurs in the rat colon as well [87]. As such, rats could be a model to examine colonic changes associated with intestinal inflammation or neoplasia. It has been shown that mutation of the APC gene induces spontaneous development of tumours along the intestinal tract of rodents [64]. Interestingly, mutations of the APC gene in rats induces localized tumour development within the colon and rectum, an observation that differs from mice whereby tumour development is generally restricted to the small intestine. Thus, the rat APC mutant gene model would be a better choice for investigating the pathophysiology of colorectal cancer (CRC) in people as compared to the mouse model [88].

Another advantage of rats compared to mice is their relatively large body size and larger intestinal tract [88]. As an example, the larger physical size of the rat allows for better experimentation with the chemical incitant TNBS, as TNBS induces more pronounced intestinal injury when administered rectally (opposed to *per os*). In as such, this procedure is much easier to perform in the larger rat model as compared to mice. Finally the larger intestinal tract of the rat also allows for more tissue to be harvested, and would allow more data to be collected, and limit the need to ‘pool’ tissue samples as is often required when harvesting tissue from mice.

Although the larger size of the rat has several advantages over the smaller mouse, in general more investigations into the pathophysiology of intestinal disease in people employ mice as the primary rodent model [89], as underscored by the utility of IL-10 knockout mice used to study colitis [90, 91]. Furthermore, murine models have a considerably higher amount of genetically altered and highly conserved inbred strains to study intestinal inflammation when compared to rats, which are often composed of outbred stains (Wistar and Sprague–Dawley rats) and contain a less conserved genetic background [89]. Despite this, the rat is still a valuable animal model to study intestinal inflammation and it is conceivable that overtime, there could be an increase in the numbers of analytical tools and reagents available for rats, making rats an even more effective animal model to study intestinal inflammation in people.

### Nematodes and insects

Nematodes are not considered a ‘conventional model’ to study mammalian intestinal inflammation; however, nematodes can provide insights into the mechanisms involved in innate immunity and defence. Although nematodes are small and lack an adaptive immune system, they share several characteristics that are similar to the mammalian intestine including: a modified innate intestinal immune system; production of antimicrobial peptides; similar signaling pathways; and a plethora of complex intestinal bacteria-enterocyte interactions [47, 92]. To exemplify this, *Caenorhabditis elegans* has been used to examine host-microbiome interactions in the intestine at the apical surface of epithelial cells [93]. In its natural habitat, *C. elegans* can be isolated from rotting fruit in the soil and is known to consume bacteria as its main food source [94]. Bacteria belonging to the *Proteobacteria* phylum act as commensal residents within the *C. elegans* intestine, and some species such as *Salmonella enterica* serovar Typhimurium, and *Yersinia pestis* have been suggested to cause injury to the nematode intestine, similarly to injury observed in the mammalian intestine [94, 95]. Notably, this intestinal model has been used to examine bacterial populations required to maintain intestinal homeostasis and to investigate mechanisms of epithelial defense [93, 95]. *C. elegans* has been used to visualize events involved in the induction of necrotizing enterocolitis through the analysis of serine and proteases inhibitor activity on epithelial cell function [47]. The pathogenicity and intestinal injury caused by *Listeria* sp., *Salmonella* sp. and *Shigella* sp. has also been investigated using the *C. elegans* model [96]. As an example, *Listeria monocytogenes* induces intestinal epithelial changes in *C. elegans* by processes that are independent of traditional bacterial translocation through goblet cells or epithelial cell junctions [97].

Similarities are also observed when comparing nematode and mammalian signalling pathways, protein secretion, and expression of transcription factors [98]. As examples, the *C. elegans* NSY-1/SEK/PMK-1 MAP kinase pathway has been identified as a mammalian MAP kinase ortholog [93], and studies examining kinase activation identified this pathway in the *C. elegans* NF-κB response parallel to mammalian innate immune system activation independent of TLR signalling [93]. Another advantage of using *C. elegans* is that it is translucent and this characteristic enables investigators to visualize real-time events involved in digestion and innate immune function [47]. This attribute has been useful in measuring temporal changes involving intestinal cell integrity, and the subsequent progression of intestinal inflammation following challenge with pathogenic *S. enterica* or *Escherichia coli* species [95, 96].

Insects possess many of the same attributes as nematodes making them a valuable model to study intestinal function. Recently, the fruit fly *Drosophila melanogaster* has been used to study the mechanisms involved in intestinal function and disease. Specifically, the *D. melanogaster* model has been used to examine changes in the innate immune response as it relates to chronic inflammation and cancer development [99]. *Drosophila* can provide a highly applicable system to study mechanistic changes in the host genome. The innate immune response of *Drosophila* is often associated with antimicrobial peptides (AMPs) and the reactive oxygen species (ROS) response produced by its epithelia, followed by immobilization of phagocytic haemocytes which engulf foreign materials [100]. Specialized *Drosophila* cytokines such as the Toll ligand, Spätzle (Spz), and unpaired 3 (Udp3) also contribute to an immune response, whereas the specialized Imd pathway responds to Gram negative bacteria and activates antibacterial peptide genes through NF-κB-like proteins [100]. Presently, cell signalling pathways involving innate immune functions have been studied in the *Drosophila* intestine. *Drosophila* possess an immune deficiency pathway that is functionally similar to the NF-κB signalling pathways in mammals, and uses the dual function NADPH oxidase (DUOX) pathway to produce ROS as a means of bactericidal defence within the intestine [101]. Importantly, these events in the *Drosophila* intestinal epithelium are mechanistically similar to defences observed in human beings; furthermore, components of the epithelial architecture of the *Drosophila* intestinal epithelium are also similar to people [100, 101]. Structurally, the epithelial monolayer and brush border, enterocytes, and crypts of *Drosophila* are also comparable to mammals [102]. Intestinal epithelial cell regeneration and differentiation in *Drosophila* is also homologous in mammalian cells, and is exemplified in the Notch, K-Ras/Ras, JNK, and Wnt/wg signalling pathways [102].

*Caenorhabditis elegans* and *Drosophila* can be used as effective invertebrate models for identifying early processes involved in the initiation and progression of innate aspects of intestinal inflammation [103], cell signalling, epithelial barrier function, and the impact of bacterial populations on intestinal physiology [47, 95]. A major limitation to the use of these invertebrate models is the lack of an adaptive immune response and some cellular processes that are present within the mammalian intestine [104]. This is underscored by the induction of intestinal injury in the *C. elegans* model by *L. monocytogenes* employing mechanisms that do not involve translocating through goblet cells or epithelial junctions, a feature that is employed within the mammalian intestine [97]. Furthermore and in general, the *C. elegans* model may not be ideal to investigate host-microbiome interactions with respect to human pathogens, as many intestinal bacteria and other microorganisms are harmful to the *C. elegans* intestine [105]. This observation is highlighted in a study that challenged *C. elegans* with either commensal *E. coli* or *Citrobacter rodentium* in the presence of *Giardia duodenalis,* which resulted in increased mortality in the treated worms. This indicates that caution should be used when employing *C. elegans* to investigate the interactions between the intestinal microorganisms and the host [106]. Finally, a potential limitation of insect models is that portions of the foregut and hindgut are lined with chitin. Certain holometabolous insects produce a peritrophic matrix that is functionally similar to the intestinal glycocalyx of mammals and serves to protect the gut from mechanical damage and also acts as a barrier against the invasion of microorganisms [107]. Importantly, the peritrophic matrix is composed of microfibrils rich in chitin, a product that is not present in the mammalian intestine [107]. Thus, insect models may not be appropriate for studying either the physiological functions of the intestinal glycocalyx or microbial interactions with the glycocalyx.

### Fish

Zebrafish (*Danio rerio*) have been used to model the human intestine for many years, and although this is a non-mammalian vertebrate model, it is a highly versatile model that provides researchers with the option to study both innate and adaptive immune responses [48]. Zebrafish are considered by many to be superior to invertebrate models as they have a larger repertoire of organs that exhibit pathologic changes. Similarly to *C. elegans,* they have a transparent embryo and larvae, relatively simple husbandry requirements, and are highly fecund [108]. The zebrafish intestine possesses similar cell types to mammals such as absorptive enterocytes, endocrine and goblet cells, a functional brush border with microvilli, and an epithelium that is continuously sloughed off into a luminal space and regenerated in a manner parallel to the murine and human intestine [109]. Zebrafish do not have a defined stomach, therefore its strengths as a nutritional model are limited as most protein and fat absorption occurs in the lower intestine rather than in the small intestine [109]. Despite this limitation, the zebrafish model has proven useful for studies of intestinal motility and peristaltic events through a mutation that leads to the loss of enteric neurons [108]. Most studies examining inflammation events in zebrafish have utilized chemical incitants, namely TNBS and DSS [110]. The zebrafish model has been well established to study host-microorganism interactions and bacterially triggered immune responses [111]. The establishment of a germ-free zebrafish model has enhanced its ability to be applied to microbiome research, and researchers have used germ-free fish to understand and compare the richness and abundance of microbial communities [112, 113]. Significantly, the adaptive immune response in zebrafish develops to maturity in approximately 3weeks, and the use of zebrafish at 3-weeks of age or younger allows researchers to study innate responses without the interference of the adaptive immune response [48]. As zebrafish possess a functional innate and adaptive immune response, their use in combination with larger animal models can be very advantageous to elucidate the role of the intestinal microbiome on enteric disease. Applications of the zebrafish model to further understand the effects of acute and chronic inflammation on intestinal cells can be very useful, and it is expected that this model will become increasingly utilized once more biomolecules and techniques are developed. The zebrafish intestine has the added advantage of being homologous to the human intestine structurally, and the ability to use direct live imaging to view the epithelial cells in real-time during infection significantly increases the effectiveness of this model.

### Pigs

The pig is an excellent mammalian model to study the mechanisms involved in acute and chronic intestinal injury and inflammation, as the intestine is anatomically and functionally similar to the human intestine [49]. The anatomic structure of the pig gastrointestinal tract, in particular the stomach and small intestine, is analogous to the arrangement in human beings and differs only by the spiral orientation of the pig colon and the lack of an appendix [114]. Despite this, primary intestinal functions such as nutrient and water absorption and microbial fermentation are still comparable to the human intestine [114, 115]. Additionally, intestinal digestive enzymes, secretory proteins and the microbiome within the pig intestine are also comparable to people, facilitating the examination of the relationship between microbial communities, diet and intestinal health [114, 116]. The pig model has also been used extensively to replicate the human microbiome in the pig intestine through fecal transplantation procedures [115]. Several studies have also examined the pre-colonization of piglets with probiotic and avirulent bacterial strains common to the human microbiome, and concluded these strains were protective against subsequent infection with pathogenic bacteria [117, 118]. Many of the immune cells and processes of the innate and adaptive immune system, namely populations of mucosal and intraepithelial B and T lymphocytes and the recognition of activators of innate immunity (i.e. LPS and nucleic acids) by macrophages, are also comparable to those in human intestinal immunity [119, 120].

Although pigs are an excellent animal model to study intestinal inflammation of people, there are variations in the porcine adaptive immune response. Most notably, the pig has an abundance of intraepithelial and plasma γδ T-cells in comparison to mice and human beings [121]. In human and murine models, it is uncommon to find mature, resting CD4^+^CD8^+^ T-cell subsets in peripheral blood [120], however in pigs, cattle and sheep these populations are highly elevated and are suggested to increase T-cell memory during infection [122]. Furthermore, the high number of γδ T-cell receptors (TCRs) in the pigs make it an effective immune model to study this T-cell lineage, as the low presence of γδ TCRs in the human and mouse models proved to be inefficient to understand how these cells rapidly respond to bacterial antigens in the host intestine [123]. Although in pigs more CD4+ T-cells also co-express CD8^+^ TCRs, similarities between the murine, human and porcine Treg response exist regarding the CD4^+^CD25^+high^ T-cell population and the increase in IL-10 expression via Foxp3 regulation in all three species [124]. Differences in immunoglobulin expression also exist; in the human immune response IFN-γ increases the expression of IgG1, whereas in swine Th1 cytokines such as IFN-γ, IL-12 and IL-10 promote IgG2 expression [125]. In addition, expression of Th2 cytokine IL-4 is downregulated in pigs, as compared to human beings and mice, which show increased expression of IL-4 when induced [119].

The use of pigs as a large animal model enables researchers to harvest large amounts of tissue, a distinct advantage when investigating intestinal inflammation. The size, slow growth rate and relatively slower reproductive rates of the pig however, are unfavourable qualities when studying intestinal inflammation relative to other animal models. For instance, commercial swine have rapid growth rates and can gain 90 kg of weight in 18 weeks [126]. These large animals also require large housing facilities and husbandry costs compared to smaller rodent and invertebrate models. Additionally, the 114 day gestation period in swine is much longer than the 21 day gestation period in mice [127], and sexual maturity is reached at 6–8 months in swine, compared to 6–8 weeks in mice [128]. Collectively, the reproductive biology, increased costs of housing and the overall size of pigs can limit their use as an intestinal inflammation model in comparison to rodents, invertebrates and fish. Despite the drawbacks (e.g. cost, husbandry challenges, spiral colonic anatomy, large cecum, and lymphocyte variations), pigs are considered to be a good large animal model to study intestinal inflammation of people.

### Non-human primates

Non-human primates (NHPs) are considered the best animal model to study the mechanisms involved in acute and chronic inflammation, as there are irrefutable similarities to human intestinal physiology, function, immunology, and the intestinal microbiome. Macaques and tamarins are most commonly used to study mechanisms involved in both the pathogenesis and treatment of intestinal disease [129]. Interestingly, these NHPs often develop spontaneous colitis and subsequent colon cancer following extended periods of confined captivity, and thus are good models to examine the de novo generation of intestinal inflammation and neoplasia [129–131]. For instance, Gozalo et al. [130] demonstrated that tamarins held captive for an average of 100 months develop terminal spontaneous ileitis that initially presents as chronic diarrhea 3–6 months prior to the confirmation of ileitis. Other researchers have shown that tamarins can develop spontaneous and chronic colitis around 2 years of age [132], and studies suggest that stress in tamarins and the ambient temperature of their housing facilities during captivity may contribute to the development of colitis [133, 134]. Although many of the cellular mechanisms involved in the development of spontaneous colitis remain unknown [135], Ramesh et al. [136] examined the cytokine production in gut-associated lymphoid tissue (GALT) of rhesus macaques exhibiting signs of persistent diarrhea. These macaques with enterocolitis had higher amounts of TNF-α and IL-1α in the GALT and non-intestinal lymphoid tissue, an observation consistent with human patients with necrotizing enterocolitis [131, 136, 137].

Non-human primates are also used to investigate the effect of microorganisms on the development of intestinal inflammation, and have demonstrated that intestinal bacteria can influence the onset of disease [138]. Importantly, the microbiome in NHPs, rodents, zebrafish, and human beings all possess similarities to one another, and imbalances made in the intestinal communities can lead to disease. Not only are the populations of the bacterial communities critical to maintaining homeostasis in the intestine, but species of Archaea within the intestine also contribute to the maintenance of a well-functioning microbiome. Investigations have identified methane-producing Archaea species and sulphate-reducing bacteria (SRB) collectively produce metabolic by-products associated with poor colonocyte health and function [41]. Furthermore, the severity of disease increased with higher amounts of SRB. As these bacteria increased in number, the hydrogen sulfide concentration in the colon also increased [139], suggesting a connection between methane-producing Archaea and SRB with intestinal health. Further, *Macaca* sp. have been used to examine the pathophysiology of *C. jejuni* infection, and it was shown that neutrophil and lymphocyte infiltration in the mucosa along with bloody stool and watery diarrhea were observed, aligning with the tissue changes of acute colitis observed in human campylobacteriosis [140]. Finally, rhesus macaque populations have also shown that species-specific *Helicobacter* spp. infections induce colitis [141], while *Shigella flexneri* infection in rhesus macaques has been associated with mucosal invasion and marked imbalances in electrolyte transport—manifestations similar to shigellosis in people [131].

The intestinal environment is in a constant state of flux and as such is either in a state of immunological quiescence or activation, and it has become clear that alterations of these states can impact brain function and the mental health of the host. As such, researchers are now using animal models to examine the effect of the intestine on mental health. Non-human primates can be employed to investigate the relationship between the intestinal microbiome, the intestinal nervous system, and the impact on host well-being. The gut-brain axis is a functional link between the intestine, the autonomic nervous system, and the higher functions of the brain. Innervation of the autonomic nervous system has shown that perturbations in the intestinal microbiome influence brain function and behavior, and thus can result in changes in feeding behavior, anxiety-like behaviour, stress, depression, and pain perception [34]. Many of these functional changes are observed in patients suffering from IBD and IBS [21, 142]. Although mice have also been used to study certain aspects of the gut-brain axis [143], NHPs likely are the best model to study neurological activity for human beings. Many NHPs characteristically develop deeper social bonds and display behaviours indicative of higher human-like intellect, giving them preference over the mouse and pig animal models for brain-related investigations.

The use of NHPs appears to be the most representative animal model to simulate intestinal inflammation in people. This model however, has considerable drawbacks. Most notably, the ethical use of highly intelligent animals closely related to human beings is controversial, and generates charged discussion between the scientific communities involved in animal research and the general public. Many people also believe NHPs such as the near extinct African Great Ape species should be banned from scientific research [144]. NHPs also require specialized housing facilities with an extensive and expensive biosecurity infrastructure, as well as elaborate equipment for environmental enrichment [145]. Moreover, several species of NHPs are large (gorillas, chimpanzees and orangutans) and can be potentially intractable and dangerous, requiring animal care staff and veterinarians with specialized training in animal husbandry, safety, and disease control. Furthermore, NHPs may carry zoonotic organisms that are highly pathogenic and easily transferable to people. One of the most well-known, potentially fatal pathogens carried by NHPs is *Cercopithecine herpesvirus* (B virus) [146], and although it is relatively innocuous in monkeys, people who are exposed to the virus through secretions from bites or scratches can develop a fatal form of viral myeloencephalitis [146].

### Other animal models

**Fig. 3:**
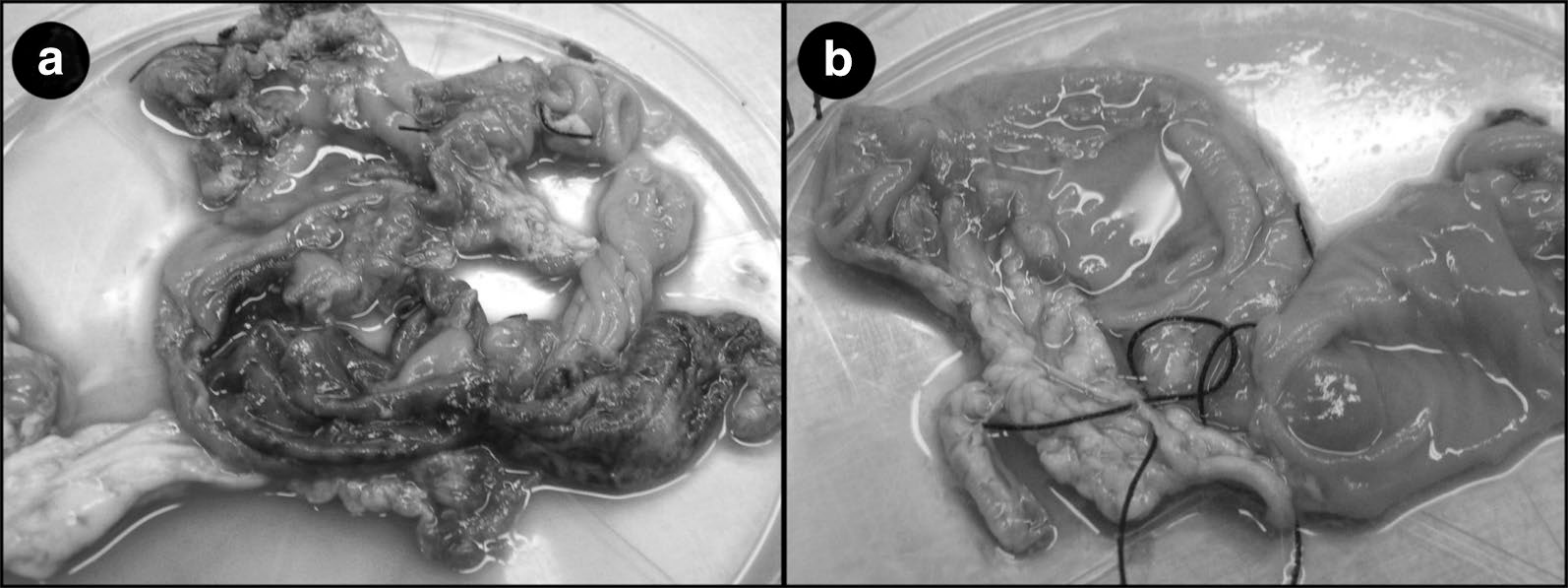
Sheep intestinal loop model. Ovine ileal segments inoculated with **a** *Campylobacter jejuni* 81-176 or **b** phosphate buffered saline and harvested 48 h post inoculation. *Campylobacter jejuni* treated intestinal loops are markedly edematous, congested, and presented with numerous fibrino-hemorrhagic foci of mucosal necrosis

There are other less frequently used animal models employed in intestinal inflammation and disease studies. Gnotobiotic juvenile beagles have been used to study colitis induced by *C. jejuni,* with results indicating mild colitis develops in the absence of mucosal infiltration [147]. More recent studies have used German Shepard dogs to study canine IBD, and attempt to make comparisons between cytokine expression in the dog intestine and alterations in gene expression observed in human patients with IBD [148, 149]. Sheep as a ruminant model have also been used for intestinal investigations, but unlike monogastric species ( *Homosapiens*, NHPs, rodents, and pigs), the majority of bacterial fermentation of carbohydrates tends to occur in the rumen and not in the large intestine [150]. As such, ruminants are not ideal models to use for microbiological and nutritional studies of intestinal inflammation in people due to the importance of the rumen in ruminant nutrition; the rumen also harbors a microbial community that differs greatly from the large intestine of monogastric animals. Most research involving nutrition in ruminants focusses on the rumen. The ruminant intestine remains an area less studied, although a few studies have utilized the fetal ovine intestine for inflammation-based research [151, 152]. Some research has suggested that the presence of *Mycobacterium avium* subspecies *paratuberculosis* (*Map*), a bacterium that causes intestinal disease in cattle, can be associated with people with CD [153]. Although most studies provide contradicting evidence regarding the presence of *Map* in CD patients, some researchers have suggested that this cattle enteric pathogen can contribute to the onset of CD in human tissue, and *vice versa* [154, 155]. The bovine animal model has also been used to study non-typhoid enteric infection induced by *S.enterica* serovar Typhimurium, since wild-type mouse models tend to develop a fatal systemic form of typhoid following challenge with the bacterium [156]. A few studies have used sheep as comparative models for human studies, using intestinal loops in neonatal sheep to study mucosal immune function [157]. Although this model is good for studying the impact of pathogens on intestinal injury (Fig. 3), this particular model was limited to examining intestinal changes in the upper small intestine, and did not examine the interactions within the lower intestine, diet (i.e. ingesta), and microbial colonization [157]. The rabbit is another animal model that has been used to study colitis. In one study, bacterial muramyl dipeptide was emulsified with Freund’s incomplete adjuvant and administered rectally into the submucosa for a period of 1 month [158]. Following muramyl dipeptide administration, mononuclear cell infiltration, lymphoid aggregation, and transmural inflammation were observed in the rabbit colon [158]. Of late, preterm rabbit models have been used as a method to understand physiologic and biologic changes associated with intestinal dysfunction, neonatal necrotizing enterocolitis, and rectal-anal obstruction [159]. Rabbits and guinea pigs have also been used to study intestinal lesions resulting from the administration of common chemical incitants (to be discussed later in the review) of intestinal inflammation [52, 56].

### Surgical models

A number of surgical models have been developed to study inflammation. Surgical models possess a multitude of advantages including the ability to manipulate physiological and microbiological processes within the intestine and circumvent some of the ethical issues encountered when working directly with human subjects. Also, surgical models deliver a number of logistical advantages including the ability to deliver and localize treatments, and to measure treatment effects in a highly prescribed manner. An example of a model that has been used to manipulate physiological and microbiological process is the cecectomy model in mice. As the cecum is a major site of complex carbohydrate fermentation in mice, surgically removing the cecum significantly alters the microbial community structure and fermentation processes [160, 161] and also affects colonization resistance. Surprisingly, this model has not been extensively used to study colonization resistance processes [160], nor has it been applied to pigs or ruminants to date.

Xenografts involve the transplantation of fetal intestinal segments from one species into a different recipient species. The recipient mice are B- and T-lymphocyte deficient (e.g. SCID, and NOD SCID gamma mice) as the use of immunodeficient mice is necessary to prevent graft rejection. The transplant tissue is implanted under the skin of the back on recipient mice, and allowed to grow. Treatments are then injected directly into the lumen of the graft via a hypodermic needle. Transplantation of human, rat, and bovine intestinal xenografts has been successfully performed [162–164], and we recently and successfully transplanted porcine fetal intestines into SCID mice. After transplantation of the xenograft, the species-specific integrity of the epithelium of the transplanted tissue is thought to be retained, however chimeric tissue can form within the submucosa and lamina propria [165]. Xenograft models are considered to be axenic, but care must be taken to avoid the introduction of contaminant bacteria during the inoculation procedure. The intestinal xenograft model has been used to measure pathologic metrics incited by a variety of biotic incitants of inflammation including *Clostridium difficile* toxin A and B [166], *Cryptosporidium parvum* [167–169], *Entamoeba histolytica* [170–173], Enterohemorrhagic *E. coli* (EHEC) [163, 174], *Helicobacter pylori* [175], *Map* [176], rotavirus [174], *Salmonella typhi* [177, 178], and *S. flexneri* [179]. Metrics of inflammation in xenografts have focused on histopathologic changes, loss of barrier function, and differential expression of pro-inflammatory genes and proteins. Many studies using xenografts have utilized uninfected controls (e.g. saline or medium). In porcine xenografts we noted that non-pathogenic *E. coli* K12 incited significant histopathologic changes that were statistically equivalent to those incited by *C. jejuni* relative to a saline control treatment (*unpublished*). In contrast, others have noted the pathogenic bacteria exacerbated the inflammatory response in xenografts relative to non-pathogenic bacterial controls [163, 176, 179]. The potential of the xenograft model as a comparative pathogenicity model is currently uncertain.

Cannulation is a commonly applied method to allow researchers to temporally obtain samples from the gastro-intestinal tract of animals. For this strategy, a fistula is established into the target region of the gastrointestinal tract, and a cannula is inserted. Cannulation is commonly used to examine nutritional metrics in ruminants (e.g. ruminal, omasal, duodenal, jejunal, ileal cannulation) [180–186], and less frequently to sample mucosa [187, 188]. The model has also been applied to monogastric animals (small intestine, cecum, colon) including rabbits [189–192], dogs [193–196], pigs [197–199], and horses [200]. The cannulation method has the advantage of allowing researchers to temporally sample mucosa, digesta or both. The salient limits of this method are the complexity of the surgical procedure, the restriction of sampling to prescribed regions of the intestine, and the inability of treatments to be localized.

The establishment of intestinal ‘loops’ through surgery generates a model to study host-pathogen interactions in a prescribed manner. Intestinal loops have the added advantage of mimicking normal intestinal physiologic, immunologic, and histopathologic responses. Importantly, treatments can be localized to a specific region of the intestine. Furthermore, treatments can be replicated within a single animal as part of the experimental design. Intestinal loop models can be divided into two basic types: recovery and non-recovery surgical procedures. For the non-recovery type, loops are established in animals under general anesthesia. This involves ligating the small or large intestine to generate a compartment or compartments (± flushing the intestinal segment to remove ingesta before generating the loops). Importantly, vascular and lymphatic functions are not disrupted by the procedure. While the animal is under anesthesia, treatments are introduced into the loop lumen via injection. After a defined period (typically not exceeding 24 h) the animal is euthanized, loops removed, and samples are collected and processed. Although non-recovery models are much more commonly used than recovery loop models, they are limited to short-term measures and this is the primary limitation of the model. Non-recovery loop models have been established in a variety of animals including rats, rabbits, sheep, and cattle [201–204]. In contrast the recovery loop model has the advantage of allowing researchers to measure treatment effects over a prolonged period (>6 months) [157]. For recovery models, the intestine is exposed in an animal under anesthesia, and a section of intestine to generate the intestinal segment within which ‘loops’ will be established is identified and ligated [205]. The intestine is then cut, and the intestinal segment designated for loops is flushed with broad-spectrum antibiotics or saline. The non-intestinal segment side of the intestine is then rejoined to form a continuous and functioning intestinal tract. Each end of the intestinal segment is closed, the segment is partitioned into ‘loops’, treatments are injected into the loops, and the abdominal cavity and muscle are closed. Animals are carefully monitored, and their recovery is uneventful. At the desired time, the animal is humanely euthanized, the compartmentalized intestinal segment is exposed, loops are removed, and samples are collected and processed (Fig. 3). The primary disadvantages of the recovery model are its technical complexity, need of surgical infrastructure, skill to successfully complete the surgical procedure, and requisite post-operative measures must be adhered including the administration of analgesics and antibiotics. The single window of opportunity to administer treatments (i.e. at the time of surgery) can also be a limitation of this model. Furthermore, samples of both the intestinal mucosa and luminal contents (e.g. sloughed mucosa within the loop lumen) can only be obtained at the termination of the project. For researchers studying bacteria and inflammatory processes, the administration of antibiotics and analgesics can directly affect the treatment itself and alter immune function, thereby confounding results. As a result, a catheterized loop model was developed in which long-term catheters were inserted into the loops [205]. Notably, the establishment of catheters in loops allowed for the introduction of multiple treatments over an extended interval, following recovery from surgery and clearance of drugs administered during surgery and the post-operative period. Furthermore, observations from loop models that have been successfully established in sheep [205, 206] and pig (*unpublished*) have suggested that there is no effect on intestinal function following establishment of the loop [206]. A limitation of this model is that use of antibiotics does not eliminate microorganisms within the loops, and sloughed mucosa within the lumen of loops can interfere with the uniform distribution of administered treatments and sample collections.

### Ethical, biosafety, and biosecurity considerations

All countries must adhere to standards for the ethical use of animals in research. In Canada, the Canadian Council on Animal Care (CCAC) is the national peer-review organization responsible for setting, maintaining, and overseeing the implementation of standards for animal ethics and care in science (http://www.ccac.ca). Animal use is permissible only if the research promises to contribute to the understanding of fundamental biological principles, or to the development of knowledge that can reasonably be expected to benefit human beings or non-human animals. Animals should only be used if non-animal alternatives do not exist. In the study of inflammation, research must involve the use of animal models (i.e. to mimic the complex host–pathogen–microbiome interaction). Animals used in inflammation based research must be maintained in a manner that provides for their physical comfort and psychological well-being, and expert opinion must attest to the potential value of studies with animals before research commences. A hallmark of inflammation is pain, and thus degrees of pain or distress are concomitant in studies of inflammation. The level of invasiveness and the procedures implemented to address this must be specified and evaluated in advance. Research studying inflammation commonly falls within invasiveness categories C (i.e. minor stress or pain of short duration) and D (i.e. moderate to severe distress or discomfort). However, in relatively rare instances, research may fall within invasiveness category E (i.e. severe pain near, at, or above the pain tolerance threshold of unanesthetized conscious animals). As pain must be minimized both in intensity and duration, research that is categorized as invasiveness category E will not be approved without strong justification. The application of quantitative pain assessments is mandatory, and any animal observed to be experiencing severe and unrelenting pain or discomfort must be humanely euthanized (i.e. alternative endpoint). Similar guidelines are in place for the United States and the European Union (http://www.ccac.ca/en_/resource-centre).

As research on inflammation commonly involves the use of biological incitants of enteritis (e.g. pathogens), research must also meet all requisite biosafety and biosecurity standards to ensure safety. In this regard, all scientific activities conducted within signatory countries that involve pathogens must adhere to United Nations conventions on biosafety and biosecurity. In Canada, biosafety and biosecurity is regulated by the Public Health Agency of Canada, and the Canadian Food Inspection Agency under separate acts. The office of Laboratory Biosafety and Biosecurity specifies the physical and operational guidelines, and the evaluation and approval processes that must be met for research involving risk group 2, 3, and 4 organisms and toxins in laboratories and animal facilities, including the importation and distribution of animal pathogens (http://www.phac-aspc.gc.ca/lab-bio/index-eng.php). The standards are specified within the Canadian Biosafety Standards and Guidelines (http://canadianbiosafetystandards.collaboration.gc.ca/index-eng.php).

### Choosing the appropriate animal model

Identifying the best animal model to study intestinal inflammation is an important consideration and requires a thorough understanding of the advantages and disadvantages of each model, as there are many factors to consider. Animal models with comparable intestinal anatomy (monogastric vs. ruminant), function, and microbiome to human beings are typically the best models to examine intestinal inflammation; swine, rodents, zebrafish, and NHPs possess many traits in common with people. Moreover, animal models that can be genetically engineered and have a similar genome to the human genome allow researchers to investigate specific genes related to intestinal disease. Most certainly, genetically modified mice have become instrumental to inflammation studies, due to their ability to display phenotypic traits definitive of specific gene manipulations. Animal husbandry is also an important factor to consider, as the cost of the facilities and equipment can be prohibitive. Moreover, some animal species such as NHPs require enhanced training by animal care personnel and specialized veterinary care and service. The availability of biologic techniques and analytical tools necessary to study intestinal function and inflammation are also important factors to consider when choosing an appropriate animal model.

Presently, there is no ‘perfect’ animal model that can address all the mechanisms involved in intestinal inflammation. Each animal model has an array of advantages and disadvantages to its use and therefore a comprehensive study examining multiple aspects of intestinal inflammation requires the use of two or more animal models. For instance, using an invertebrate model to study mechanisms involved in innate immunity in conjunction with a genetically engineered murine model could provide a broader understanding of the causes of intestinal inflammation with respect to both innate and adaptive immunity. Alternatively, a mouse model can be used to determine the immunologic mechanisms of pathogen challenge on the intestine, and these observations paired with the effects of the pathogen on intestinal architecture and enterocyte function in the swine or NHP. Importantly, researchers must proactively consider the advantages and disadvantages of each model and determine the most suitable model(s) to address specific aspects of intestinal inflammation being investigated (Table 3).

**Table 3:**
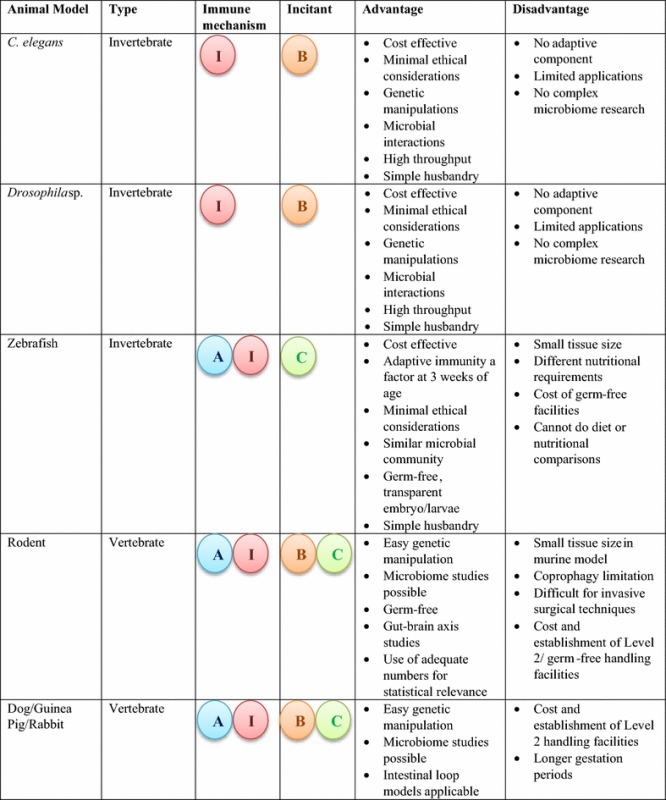

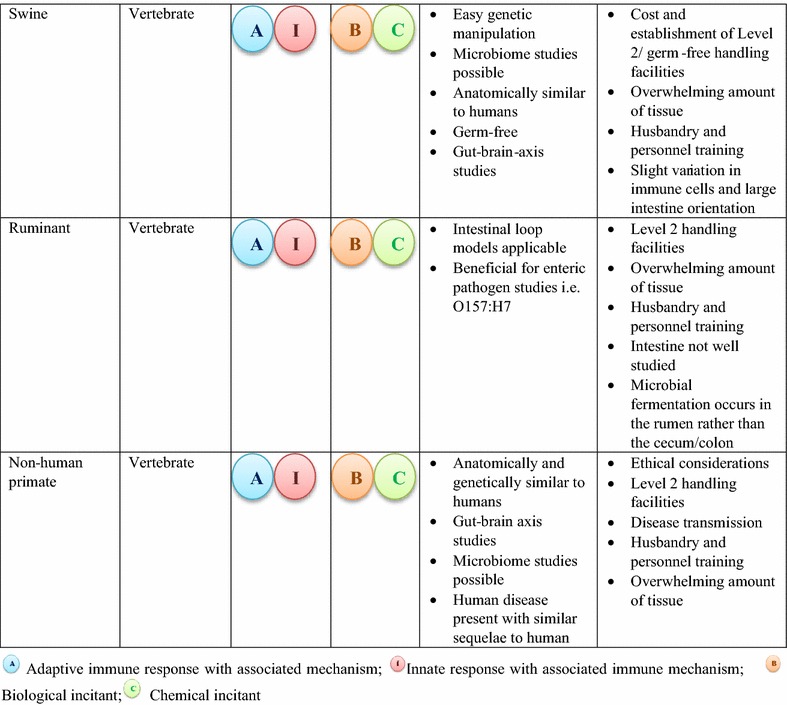
Advantages and disadvantages of various animal models used to study intestinal inflammation based on the immune response elicited by biological and chemical incitants

### Methods to incite intestinal inflammation

The induction and subsequent progression of intestinal inflammation is a complex, multifactorial interaction between the host and its environment. In particular, the physiological status of the host plays an important role in the onset and severity of disease, and as such, prior use of products such as antibiotics can contribute to the establishment of pathogenic transient bacteria in acute inflammation [207]. Other factors such as age or genetic predisposition can also contribute to inflammatory disease development in chronic disease [18]. Although the onset of intestinal inflammation can occur spontaneously in various models [208, 209], the use of either chemical or biological incitants can be effective in inducing a rapidly developing and robust response. The array of chemicals available to induce intestinal inflammation permits one to choose between acute and chronic intestinal responses, and some chemicals have the potential to incite both forms of inflammation. Similarly, various bacterial agents can induce both acute and chronic forms of intestinal inflammation. Chemical and biological incitants of inflammation are necessary to simulate inflammation in appropriate models and often closely representative diseases may not be obtainable using a single model, necessitating the use of multiple models to accurately study the disease. Similar to choosing the best animal model to investigate specific aspects of intestinal inflammation, choosing the most effective chemicals and biological agents to incite inflammation must be carefully considered.

### Chemical incitants

In animal models of inflammation, chemicals are often used as fast, economic and effective strategies to induce intestinal tissue injury. Animal models of colitis frequently use either DSS or 2,4,6-trinitrobenzene sulfonic acid (TNBS) to initiate inflammation. Acetic acid, oxazolone, and azoxymethane (AOM) have also been used, but to a lesser extent than DSS and TNBS (Table 4). The effectiveness of inducing tissue injury following treatment with chemical agents varies and depends on the molecular weight, concentration, manufacturer, and batch of the chemical [210]. In addition, the species, gender [56], and the genetic background of the animal model being challenged influences the degree of tissue injury [211, 212]. The method of administration also influences the induction and severity of disease, as some chemicals work well to induce inflammation after ingestion [56], while others function best when applied directly to the site of infection, such as the rectal administration of haptenating agents [132]. Furthermore, microorganisms present in the intestine can interact with the chemical incitant and interfere with its ability to effectively incite tissue injury [213]. In general, chemical incitants induce tissue damage that can effectively represent clinical cases of intestinal inflammation.

**Table 4:** Chemicals used to incite acute and chronic intestinal inflammation

Chemical	Effect	Disease-like symptoms
Dextran sulphate sodium (DSS)	Extensive basal crypt and epithelial cell damage with long-term administration, increase in IFN-γ, IL-6, TNF-α, IL-4 and IL-5; Th1 and Th2 mediated immune response [59]	Acute mucosal and vascular injury in low concentrations, develops in UC-like symptoms
Trinitrobenzene sulfonic acid (TNBS)	Th1 mediated immune response, ↑IL-12, TNF-α and IFN-γ [212]	Initial effects are acute in nature, develops into CD-like symptoms
Oxazolone	Th2 mediated immune response, ↑IL-4, IL-5 and IL-13 [211]	Initial effects are acute in nature, develops into UC-like symptoms
Azoxymethane (AOM)	Used in conjunction with DSS, tumour inducing, can lead to increased IL-21, IL-17A and IL-6; Th1/Th17 mediated immune response [298]	Colorectal cancer-like symptoms when used in conjunction with DSS

### Dextran sulphate sodium

Dextran sulphate sodium is used either independently or in conjunction with other chemicals to induce inflammation. By adjusting the concentration and duration of DSS treatment, the mechanisms involved in both acute and chronic inflammation can be studied. As an example, chronic inflammation in mice can be attained by administering DSS for 2 months in cycled rotations of 1 week of DSS treatment followed by 2 weeks of rest [211]. In contrast, DSS orally administered at a concentration of 1–5 % for approximately 1 week induces acute inflammation in the intestine [56, 214]. In general, DSS incites inflammation by disrupting the epithelial barrier, causing vascular and mucosal injury through the exposure of the lamina propria to luminal contents and bacterial antigens [215]. This exposure triggers the activation of inflammatory pathways resulting in an increased production of the inflammatory cytokines, TNF-α, IL-1β, IL-6, IL-10, IL-12 and IFN-γ [215]. Studies also show that in mice, expression of integrin-α M (ITGAM), integrin-α X (ITGAX), and IL-17 is increased following DSS treatment [216]. Long-term treatment with DSS increases IL-4 and IL-5 expression, suggesting that DSS induced colitis is mediated by both Th1 and Th2 immune mechanisms [215]. Furthermore, the factors involved in innate immunity are also affected by DSS treatment, as treatment with DSS changes the expression of MyD88, TLR4 and TLR9, and small changes in these mediators of innate immunity contribute to epithelial cell damage and subsequent intestinal inflammation [56].

Many factors affect the propensity of DSS to induce inflammation in different animal models. The bacterial population within the colon is a critical factor in modulating the severity of the tissue response caused by DSS treatment. This is observed in DSS treated germ-free mice, which develop severe colitis upon treatment with 1 % DSS, whereas conventional mice with an intestinal microflora showed only minor intestinal crypt damage and a relatively non-severe colitis with the same treatment [213]. The ability of DSS treatment to induce intestinal inflammation is also affected by the genetic background of the animal species. For instance, C3H/HeJ mice are sensitive to DSS treatment, whereas C57BL/6 mice are relatively resistant [56]. For instance, C3H/HeJ mice treated with DSS have greater occurrence of bloody diarrhea, epithelial ulceration, inflammation, and weight loss as compared to C57BL/6 mice [56, 217]. In addition to mice, pigs have been used to examine DSS induced intestinal inflammation. Young et al. [218] observed increased expression of TNF-α, IL-6, IFN-γ, and IL-17A in pigs administered DSS, an observation that is similar to clinical patients suffering from active IBD. Other studies in pigs report increased lymphocyte infiltration in mucosal tissue as well as mucosal erosion and crypt destruction following DSS treatment, and that these tissue changes are similar to the intestinal lesions often present in people afflicted with IBD [219].

Although DSS is an effective inducer of intestinal inflammation, there are potential drawbacks to its use in animal models. Most notably, there can often be significant inter-animal variability in the severity of tissue injury. In many cases, marked inconsistency in the amount of mucosal damage and in particular epithelial cell necrosis is observed [56, 210]. Furthermore, the molecular composition and purity of the chemical product can vary between the product batches and chemical supplier, potentially leading to inaccurate concentrations and volumes of DSS administered to the test animals [211]. Although the level of tissue injury can vary between treatment groups with DSS, it is still considered a model chemical incitant of intestinal injury and is commonly used to stimulate UC-like lesions in various animal models.

### Azoxymethane

The severity of inflammation can be enhanced by administering chemical incitants of inflammation in combination with another chemical inducer of inflammation. Long-term administration of DSS with AOM induces chronic intestinal inflammation that often progresses to CRC [220]. Notably, the intestinal lesions induced by DSS with AOM treatment are consistent with intestinal changes manifested by patients with UC [59]. Treatment with these two chemicals alters tissue cytokine profiles in mice resulting in increased expression of IL-4 and IFN-γ, parallel to expression in patients with UC [59]. Furthermore, the administration of AOM with DSS is necessary to exacerbate the effects of DSS to induce the development of colorectal cancer [221], an event that can on occasion occur in people with UC [2]. Thus, the combined use of both chemicals is ideal for investigating both inflammatory diseases of the intestine, as well as the pathophysiology of colorectal neoplasia.

The administration of AOM alone has also been used to study mechanisms that induce cancer in the distal colon [222]. Proposed mechanisms for the induction of inflammation and tumour formation by AOM include the upregulation of cyclooxygenases leading to the enhanced production of prostaglandin E_2_ [220], and the induction of pro-mutagenic epithelial changes caused by the O^6^ methylation of guanine to induce tumour formation [223]. Several other metabolic pathways are also affected by AOM metabolism, including the k-ras regulated MAPK intracellular signalling pathway, the cellular adhesion related B-catenin pathway, and the epithelial cell apoptotic TGF-β pathway [222].

### Trinitrobenzene sulfonic acid

Trinitrobenzene sulfonic acid is primarily used to establish acute intestinal inflammation in animal models, but can also be employed to induce chronic inflammation in rodents [79, 224], pigs [225], rabbits [56], guinea pigs [52], and NHPs [226]. To become chemically active, TNBS needs to be solubilized in ethanol, and this TNBS-ethanol mixture induces intestinal inflammation by altering host proteins through the formation of covalent bonds with trinitrophenyl haptens of TNBS [132]. This process stimulates an immune-mediated inflammatory response. The TNBS-ethanol mixture produces ‘hapten modified self-antigens’ that are recognized by the host immune system and contribute to acute intestinal inflammation [132]. Moreover, ethanol also acts as an irritant that contributes to the damage of the epithelial barrier [214]. Treatment with TNBS-ethanol can also produce intestinal lesions representative of those present in individuals with IBD [72]. It has also been shown that rectal administration of TNBS in 40–50 % ethanol leads to colon shortening, intestinal hemorrhage, epithelial necrosis causing crypt architecture destruction, and transmural inflammation accompanied by an elevated Th1 immune response within the colon [214, 227].

Trinitrobenzene sulfonic acid can be used as an incitant of both acute and chronic inflammation. In acute inflammation of mice, the primary immune response observed follows a pro-inflammatory Th1 response, increasing the expression of IL-12, IFN-γ and TNF-α [214, 228]. An increase in IL-23 production indicates the initiation of chronic colitis in BALB/c mice, and as colitis progresses, the cytokine profile changes to a Th17 dominant response, evident by increased IL-17 and IL-25 expression, before ultimately switching to an IL-13 dominant immune response [228]. Research also suggests that a T-cell deficient mouse exhibit chronic enteric inflammation in the presence of IL-23, making this cytokine a determinative marker for chronic enteritis [229]. Importantly, these observations are aligned with cytokine profiles in CD patients as these individuals display elevated levels of IL-17, IL-22 and IL-23 [230, 231]. Rats supplemented with TNBS often lose weight, present with bloody diarrhea, and exhibit marked mucosal and transmural intestinal inflammation, similarly to people with IBD [232].

Although intestinal inflammation has been established in rodents, swine, and NHPs using TNBS as the chemical incitant [52, 56], evidence indicates that mice are the best models for investigating TNBS-ethanol induced colitis [233]. When selecting the most appropriate mouse strain to examine TNBS induced tissue injury, the genetic background and phenotypic profile of the mouse are important factors to consider. As examples, C57BL/6 and DBA/2 strains are relatively resistant to treatment with TNBS, whereas SJL/J, C3HeJ and BALB/c mice produce significant tissue injury following exposure to TNBS [132].

### Oxazolone

Oxazolone is an alternative chemical agent that can be used to produce ‘hapten-like proteins’ in the host intestine to induce acute intestinal inflammation [234]. Its use results in intestinal lesions associated with a predominant Th2 immune response. The tissue lesions manifested in mice following exposure to oxazolone are similar to UC-like lesions in people, with most lesions causing mucosal ulceration, submucosal edema, and tissue hemorrhaging [234]. In mice, oxazolone administration has been attributed to body weight loss, diarrhea, ulcers, and loss of epithelial cells in the large intestine [211, 234]. One of the advantages of using oxazolone to induce tissue injury is the rapid progression of tissue architecture alteration in comparison to other chemical agents [234]. Indeed, the relatively fast induction of tissue damage makes oxazolone an ideal candidate to study UC-like disease in mice, as histological evidence shows an increase in IL-4, IL-5, and IL-13, cytokines that are indicative of a Th2 immune response [211]. Similarly to TNBS and DSS , the choice of mouse strain will influence the effects of oxazolone treatment on tissues. As an example, oxazolone treated BALB/c mice show increased tissue injury when compared to C57BL/6 mice under the same treatment [234]. Although oxazolone is an effective inducer of acute inflammation, its effectiveness to induce chronic inflammation remains undetermined, as few investigations have examined its potential to cause long-term intestinal inflammation [234, 235].

### Chemical incitation of intestinal injury

Chemical incitants induce tissue injury by initially disrupting the epithelial barrier, exposing the lamina propria to intestinal contents, and stimulating pro-inflammatory cytokine activity. Dextran sulphate sodium, AOM, TNBS, and oxazolone cause tissue injury within the intestine, and have been especially effective in inducing injury within the distal colon [211]. Each incitant has the ability to induce distinct tissue lesions accompanied by specific helper T-cell cytokine cascades during inflammation. Individually, these chemicals are all effective inducers of disease-specific injury. For instance, DSS is very useful as a chemical model for UC-like intestinal injury [236, 237], whereas oxazolone provides the benefit of quick injury development and rapid tissue damage compared to the other three chemical agents [228]. If developing chronic inflammation in the intestine is the main focus of study, then TNBS and DSS are the most appropriate chemicals to use. In summary, TNBS, DSS, AOM, and oxazolone are all useful chemicals to induce intestinal inflammation in animal models, and the best chemical agents to employ depends on the specific aspect of intestinal inflammation under investigation. Chemical incitants are the most common agents used to induce intestinal injury and inflammation, and are often considered the best methods to study the immune response associated in intestinal disease. Chemicals agents are an inexpensive [47], quick, and effective method to cause inflammation, and these agents are valuable tools in the armamentarium for investigating the pathophysiology of intestinal inflammation.

### Biological incitants

As an alternative to using chemical incitants, biological incitants have also been used to study common intestinal inflammatory diseases. Biological incitants can be bacterial, viral, protozoal, or helminthic, and can be used to induce both acute and chronic inflammation. Herein we review the most commonly used biological agents to induce intestinal inflammation in animal models.

### Bacteria

The host intestinal tract contains a diverse community of bacteria totalling 10^13^–10^14^ bacterial cells [238], with species most often belonging to the *Bacteroidetes*, *Firmicutes*, *Actinobacteria*, *Spirochetes*, and *Proteobacteria* phyla [239, 240]. Homeostatic interactions between the host and the resident microbiome occur in the intestine , and changes in bacterial species abundance can potentially lead to intestinal inflammation [240]. It has been well investigated that the commensal bacteria are important in maintaining a healthy intestine by preventing the overgrowth of pathogenic microorganisms, and assisting in regulating and maintaining a quiescent intestinal immune system [41]. An uncontrolled immune response to commensal bacteria can lead to intestinal injury, and reports indicate that the development of aberrant immune responses can occur from increased exposure to the commensal bacteria [25, 239]. Moreover, modifications to the community structure of the intestinal microbiome can incite disease, often by the uncoordinated expression of pro-inflammatory cytokine profiles in concert with the simultaneous loss of anti-inflammatory signalling [239, 241]. A well characterized model for studying acute inflammation involves using *C. rodentium*, a attaching and effacing bacterium that colonizes the cecum and large intestine of mice [242–244]. Infection with *C. rodentium* in susceptible mice is relatively short-lived, and because peak infection is observed around day 14 with clearance by day 28, this bacterium is more suitable for studying acute mechanisms of inflammation [241]. Although *C. rodentium* lesions model acute inflammation, its infection produces both ulcerative and proliferative intestinal lesions that represent those identified in patients with UC, including dysplastic changes associated with intestinal carcinomas [245]. Importantly, mice have been used to study the progression of tissue injury, and to identify the temporal relationship in cytokine expression by four different T helper CD4 T-cells subtypes (Th1, Th17, Th2, and Treg) [246]. As infection progresses, the cytokine profile changes to a Th17 dominant response, evident by the increased expression of pro-inflammatory cytokines IL-17 and IL-22. Although 21 days is not usually considered chronic, this trend was noted in mice challenged with *C. rodentium*, and initiated with a prominent Th1 immune response that slowly switched to a Th17 dominant response near the clearance of infection around day 21 [246].

*Citrobacter rodentium* also serves as an alternative mouse model to study the virulence mechanisms related to EHEC and Enteropathogenic *E. coli* (EPEC) infection that use attaching and effacing lesions to attach to and remodel the intestinal epithelium for bacterial entry and damage [247]. A prominent EHEC serotype is O157:H7; this serotype of *E. coli* is naturally found in cattle and other ruminants, and has been responsible for numerous foodborne related illnesses, and hospitalizations and deaths around the world [248]. Pathogenesis is associated with the presence of the locus of enterocyte effacement (LEE) pathogenicity island, which is responsible for the production of the Type 3 secretion system, Shiga toxins, Tir, intimin, and enterohemolysin [248]. *Citrobacter rodentium* is also a LEE positive organism and utilizes attaching and effacing lesions to facilitate infection; however, it does not produce a Shiga toxin, meaning the tissue damage observed is not hemorrhagic , but displayed as transmissive colonic hyperplasia allowing the passage of immune cells into the lumen of the colon [247]. A number of small and large animal models have been used to study EHEC and EPEC colonization and pathogenesis including mice, rats, rabbits, pigs, cows, dogs, baboons, and macaques [249]. No single animal model can manifest lesions that are representative of EHEC-associated disease observed in people, as such using multiple animal models is a better strategy to understand intestinal inflammation in people caused by EHEC.

*Helicobacter pylori* are gram negative bacteria associated with the development of gastric ulcers in people. *Helicobacter* spp. can occupy the gastric epithelium, the intestine, and the liver in afflicted individuals [250], and have been known to colonize a variety of animals including pigs and opossums, as well as non-mammals such as tortoises and birds [251]. Research groups are now investigating a link between *H. pylori* infection and IBD [252–254]. Urease positive *Helicobacter* spp. found in the stomach and proximal small intestine of hosts, have been suggested to cause damage to the ileum and colonic mucosa with cytotoxins released in the presence of urease, and its these products that appear to contribute to the development of UC and CD-like symptoms in humans [250].

The genetics of the animal model used has a significant effect on the pathophysiology of bacterial induced intestinal injury. For example, C57BL/6 mice will develop a predominant Th1 mediated cellular response resulting in extensive epithelial cell injury and cell proliferation when infected with small numbers of *H. pylori* and *H. felis* [255]. In contrast, BALB/c mice will develop a Th2 mediated cellular response with minimal intestinal injury following challenge with large numbers of *H. pylori* and *H. felis* [255]. Studies show that non-*H. pylori* helicobacters cause IBD-like conditions in animal models, and paradoxically, research shows *H. pylori* can reduce the development of IBD in people with repeated *H. pylori* infections [256]. In such individuals, increased levels of Foxp3 and reduced intestinal inflammation have often been observed [256, 257]. Experimental colitis leading to cancer has also been induced using *H. bilis* and *H. hepaticus* in mice 4–6 weeks post-infection, and can contribute to the development of chronic intestinal inflammation in mice [258, 259]. *Helicobacter hepaticus* is often isolated in the livers and colons of infected mice [260], and induces hepatitis, enteritis, typhlocolitis, and IBD-like tissue injury in many genetically modified mouse models [64, 261]. *Helicobacter hepaticus*-induced enteric inflammation has been observed in A/JCr, BALB/cAnNCr, SJL/NCr, C3H/HeNCr, Rag^−/−^, IL10^−/−^, and SCID mice reconstituted with CD45RB^high^ T-cells [262]. Challenge studies with *H. hepaticus* in these models will develop CD-like lesions and are used to investigate mechanisms involved in the development of IBD in people [263]. As more information on the mechanisms involved in tissue injury caused by *H. hepaticus* are known, the use of this agent to study intestinal inflammation is expected to increase.

Investigations show *S. enterica* serovar Typhimurium can be used as a chronic model of intestinal inflammation in mice. This bacterium invades mucosa 27 weeks after infection and breaches the epithelium leading to the development of tissue injury in the deeper layers of the intestine [264]. A breach in the epithelium can also act as a conduit for prolonged mucosal translocation of the bacterium. *Salmonella enterica* serovar Typhimurium is used extensively in research as a non-typhoidal *Salmonella* infection model, which most often causes a non-septicemic form of enterocolitis in cattle and humans [207]. Mice are relatively resistant to developing typhoid-like lesions following exposure to *S. enterica* serovar Typhimurium [207], however, the severity of the lesions can be markedly enhanced by antibody treatment. This phenomenon suggests that disruptions in the intestinal microbiome can influence the effects of *S. enterica* serovar Typhimurium in the murine and bovine intestine [72, 156]. Further, pre-treatment with streptomycin in mice produces intestinal damage that includes epithelial crypt loss, mucosal erosion, and neutrophil infiltration that are similar to lesions observed in people with UC [72, 207].

Choosing a proper animal model is an important consideration for studying  salmonellosis. As examples, exposing LTβR knockout mice to *S.enterica* serovar Typhimurium produces acute and marked intestinal lesions comparable to human IBD lesions. These lesions can be accompanied by edema, disruption of the villus architecture, goblet cell depletion, and increased ICAM-1 expression [207]. Murine lesions also show striking similarities to *S. enterica* serovar Typhimurium infection in calves, NHPs, and people [207]. Similarly, iNOS^−/−^, IL-10^−/−^, and CD40L^−/−^ mice challenged with *S.enterica* serovar Typhimurium also develop enterocolitis [265]. Finally, *Salmonella* spp. are intracellular pathogens, and clearance of disease requires a coordinated Th1 and Th17 immune response [266]. As such, mice and NHP models have been used to investigate the expression of Th1 cytokines TNF-α, IFN-γ, IL-12, IL-15, and IL-18 [267], as well as the Th17 cytokine IL-17 in association with intestinal inflammation [268].

*Mycobacterium avium* subspecies *paratuberculosis* is the leading cause of Johne’s disease in cattle, and causes weight loss, diarrhea, and mortality in ruminants [153]. Indeed, intestinal changes in ruminants with Johne’s disease are similar to intestinal changes present in people with CD [154]. As well, *Map* has frequently been found in the environment of infected herds. Individuals with CD who have been in close contact with or consumed milk from infected cattle also present with *Map* [153, 269]. Research suggests *Map* is a useful associative model to investigate CD in people [153, 269, 270]. Experiments in mice demonstrate that *Map* can induce intestinal lesions following exposure that are highly similar to those changes seen in individuals with CD [271]. As examples, beige/*SCID* mice treated with *Map* have breaches in the small intestine mucosal barrier, and marked thickening of the intestinal lamina propria associated with the infiltration of bacteria laden epithelioid-macrophages [270]. Infections with *Map* have also been identified in captive rhesus macaques with chronic diarrhea and intestinal injury similar to lesions that are present in ruminants [131, 272]. Collectively, these observations suggest *Map* is a good associative model used to study intestinal disease in people.

The above information suggests that *C. rodentium*, *Helicobacter* spp*., S. enterica* serovar Typhimurium, and *Map* can be used as bacterial agents to induce and maintain acute to chronic intestinal inflammation in different animal models. Importantly, these models appear to represent intestinal changes observed in people with intestinal inflammatory disease. Similarly to other incitants of inflammation, these bacteria can be employed not only as primary inducers of inflammation, but also used in concert with other agents (i.e. chemical irritants), providing an effective ‘challenge regime’ to study the mechanisms involved in induction and progression of intestinal inflammation.

### Helminths

Helminths (i.e. flukes, tapeworms, and roundworms) can cause extensive intestinal inflammation and injury in people and are primarily used to investigate Th2 mediated inflammation in the intestine [273]. Parasites primarily induce the expression of Th2 (IL-4, IL-5, IL-13) and Treg (IL-10, TGF-β) cytokine profiles in conjunction with eosinophil-associated tissue inflammation [274]. Nematodes (i.e. roundworms) are the most commonly used helminth model to study intestinal disease, and *Trichuris muris* is the most frequently used nematode to incite intestinal inflammation in rodents [273]. The *T. muris* murine model induces an acute immune response characterized by the loss of barrier function in the cecum and proximal colon [275]. In addition, *T. muris* elicits a strong Th2 T-cell response following exposure to large amounts of parasite eggs, and this agent can produce intestinal lesions similar to those observed in mice treated with oxazolone [273]. In mouse models such as C57BL/6 and BALB/c mice, infection results in an upregulation of Th2 cells that secrete IL-4, IL-5, and IL-13, and increase epithelial cell turnover and permeability [273, 276]. Interestingly, although helminths primarily produce a Th2 immune response in the mouse intestine, *T. muris* induces a Th1 mediated response associated with increased levels of IL-12, IL-18, and IFN-γ in AKR/J mice [276]. This event, however, fails to clear the parasite and facilitates the development of a chronic intestinal infection [273], making *T. muris *infected AKR/J mice a useful chronic inflammatory incitant model.

The severity of inflammation observed after exposure to helminths varies depending on the genetic background of the animal model. The progression of tissue injury and the type of immune response developed can be affected by the number of eggs administered. For example, *T. muris* treated mice develop a strong Th2 response to high loads of parasite eggs (>150 eggs). In contrast, AKR/J mice develop a significant Th1 response following treatment with low numbers of parasite eggs (<15 eggs) over a 36 day period [277]. Importantly, *T. muris* infection in AKR/J mice is associated with enterocyte hyperplasia, and decreased mucin secretion [278, 279], observations that are also evident in patients with IBD [280]. In conclusion, the *T. muris* model is a proficient model to study either early Th2 acute inflammatory responses (C57BL/6 and BALB/c mice) or Th1 induced chronic intestinal inflammation in the AKR/J mouse model.

### Protozoa

*Toxoplasma gondii* has also been used in mouse models to promote intestinal inflammation. *Toxoplasma gondii* infection in susceptible mouse strains is able to produce a robust Th1 associated pro-inflammatory response in the small intestine [281]. This organism has three pathogenic strains, and of these, strain 2 is considered particularly pathogenic in people, and is able to elicit a strong Th1 cytokine response resulting in increased IL-12 and IFN-γ expression [281]. Other studies have shown that *T. gondii* infection can induce the production of IL-22, an effector cytokine associated with activated Th17 cells [282]. Furthermore, administration of low numbers of protozoan cysts (20–50) to C57BL/6 and SCID mice incited prolonged episodes of enteritis [282]. In C57BL/6 mice the chronic intestinal lesions were characterized by prominent infiltrates of macrophages into the lamina propria, contributing to ileal inflammation and mucosal necrosis, an observation similar to patients with CD [283]. This suggests that *T. gondii* is a useful agent to study chronic intestinal inflammation in mice.

### Viruses

Presently, there are few studies that conclusively demonstrate a direct link between viral infections and intestinal inflammation. Most of these studies show only an observational relationship between the induction of intestinal inflammation by viruses and the onset of IBS or IBD in people [284]. Most studies examined coincidental associations between the presence of IBD in patients, the existence of viral pathogens and their remnants (i.e. genomic DNA) within the intestine [285], and the ability of viruses to exacerbate pre-existing disease [286]. One example is the link between early onset childhood measles and the subsequent development of intestinal disease in which infants with previous history of measles-associated-pneumonia, diarrhea, and weight loss developed CD or UC later in life [287]. Another example is the relationship between paramyxovirus and Epstein–Barr virus and the development of IBD, where either remnants of the virus or lymphocytes infected with viral particles, respectively, are present in the intestine [24, 288]. Although these studies do not conclusively prove viral infections induced intestinal inflammation in healthy people, it is possible that viruses can readily affect immunosuppressed individuals. Studies in immunocompromised NHP animal models and immunocompromised people with UC and CD demonstrate that intestinal inflammation and injury can be both induced [289] and exacerbated [24, 290, 291] following exposure to viral pathogens. Mouse models have also been used to facilitate acute colitis using cytomegalovirus to exacerbate DSS-induced colitis [292]. From the information described, it appears that a causal association between viral infections and the induction of intestinal inflammation in immunocompetent individuals remains undetermined. It suggests, however, that immunosuppressed individuals and animal models are more susceptible to the development of virally-induced intestinal inflammation. In as such, viruses may only be an effective tool to investigate the mechanisms involved in intestinal inflammation in immunodeficient animal models.

### Comparison of biological incitants

Biological incitants offer the advantage of being able to study both acute and chronic inflammation using agents that naturally cause inflammation in human and non-human animal tissues. A plethora of biological incitants exist that can be applied to examine inflammation; most of these agents have been investigated and are known to cause infection in the intestine of mammals. Bacterial incitants such as *Salmonella* spp. and *Helicobacter* spp. have been used to mimic intestinal infection in animal models, and much is known about their mode of infection through information attained from human infections. Most of these incitants are best used when examining the effects of acute inflammation, however helminth and protozoan models are better suited for chronic inflammatory studies. Long-term enteritis can be induced in susceptible murine models using low levels of *T. gondii* eggs [282], whereas *T. muris* has the ability to facilitate Th1 mediated chronic inflammation in AKR/J mice [277]. The use of other agents can further enhance the effect of *T. gondii* on intestinal injury. For instance, Stoicov et al. [293] demonstrated that co-infection with *H. felis* and *T. gondii* induce significant mucosal damage and chronic inflammation in BALB/c mice. The resulting lesions are associated with a prominent Th1 immune response and muted Th2 immune response that consequently develops into long-term injury to the upper gastrointestinal tract mucosa [293]. Although *Map* and viral incitants have not been definitively associated with the onset of intestinal inflammation, the ability of these microorganisms to be either co-isolated with afflicted individuals or to exacerbate infection suggests a functional role for *Map* and viral agents in IBD immunity [24, 153, 290]. As the human intestine is occupied by many bacterial species that are critical to intestinal function and homeostasis, the use of models that can replicate this diverse and complex relationship yet allow alterations (e.g. dysbioses) in this community are best for studying intestinal inflammatory diseases. Utilizing bacterial species that cause damage in both human and non-human animal intestines allows for comparable experimental conditions that can help in understanding dysbioses in relation to inflammatory bowel diseases.

## Conclusions

As the prevalence of intestinal inflammatory diseases continues to increase, it is becoming increasingly important to elucidate causes and possible mitigation strategies. Intestinal disease can arise from a variety of factors, and the complex interactions between the host and the intestinal microbiome make determining the mechanisms involved in the induction and progression of disease challenging. Currently, a variety of animal models can be used to study the processes involved in intestinal inflammation, however, rodent models and in particular genetically engineered mice are the primary models used to study acute and chronic intestinal inflammation. The ability to modify the genetic background in mice allows specific questions to be addressed, and importantly, the information from mice can be compared to other animal models and extrapolated to human beings. The generation of spontaneous, long-term intestinal inflammation can be a lengthy process, so the use of chemical and bacterial incitants to expedite the process is often necessary. Each incitant of inflammation has the inherent ability to develop specific manifestations of tissue injury as well as corresponding immune responses within various animal models, and as such, determining which agent (chemical vs. biological) to use requires careful consideration. Furthermore, the association between the intestinal microbiome and the host adds another level of complexity to the pathobiology of intestinal inflammation. The use of animal models, appropriate chemical and/or biological incitants, and eventually applicable analytical tools are all required to study inflammation within the intestine. Together, these components facilitate our understanding into the mechanisms involved in the pathophysiology of intestinal disease and potentially set the foundation for the development of mitigation strategies that can treat intestinal inflammation in people.

## Abbreviations

IBD: inflammatory bowel disease
IEC: intestinal epithelial cell
UC: ulcerative colitis
CD: Crohn’s disease
CRC: colorectal cancer
NF-κB: nuclear factor kappa B
TNF: tumour necrosis factor
IFN: interferon gamma
Th: helper T-cell
Tfh: T follicular cells
Treg: regulatory T-cell
CD4/8/25: cluster of differentiation 4/8/25
IL: interleukin
Foxp3: Foxhead box protein 3
rRNA: ribosomal ribonucleic acid
mRNA: messenger ribonucleic acid
DNA: deoxyribonucleic acid
PCR: polymerase chain reaction
DSS: dextran sulphate sodium
TNBS: trinitrobenzene sulphonic acid
AOM: azoxymethane
GALT: gut-associated lymphoid tissue
M cell: microfold cell
TLR: toll-like receptor
NOD: nucleotide-binding oligomerization domain
LPS: lipopolysaccharide
B-cell: bone marrow derived cell
T-cell: thymus derived cell
Ig: immunoglobulin
APC: antigen presenting cell
AMP: antimicrobial peptide
ROS: reactive oxygen species
PAMP: pathogen-associated microbial pattern
NK: natural killer cell
ICAM: intracellular adhesion molecule
SCID: severe combined immunodeficiency
Spz: Späzle
Udp3: unpaired 3
TCR: T-cell receptor
CCAC: Canadian Council on Animal Care
ITGAM: integrin-α-M
ITGAX: integrin-α-X
iNOS: nitric oxide synthase
LTβR: lymphotoxin beta receptor
MyD88: myeloid differentiation primary response gene (88)
APCmin: APC gene, multiple intestinal neoplasia
RAG: recombination activating gene
ATG16L1: autophagy-related protein 16-1
CARD15: caspase recruitment domain containing protein 15
DUOX: dual function NADPH oxidase
SRB: sulphate-reducing bacteria
NHPs: non-human primates
EHEC: Enterohemorrhagic *Escherichia coli*
EPEC: Enteropathogenic *Escherichia coli*
LEE: locus of enterocyte effacement
ACTH: Adrenocorticotropic hormone
JNK: c-Jun N-terminal kinase
